# On the mechanism of hypomagnesemia with treatment-resistant seizures caused by variants of the Na^+^,K^+^-ATPase α1 subunit (ATP1A1)

**DOI:** 10.1085/jgp.202513959

**Published:** 2026-06-30

**Authors:** Nicolas Colmano, Daniel Self, Hang N. Nielsen, Rikke Holm, Chai C. Gopalasingam, Ryota Fujimura, Kazuhiro Abe, Bente Vilsen, Sho T. Yano, Pablo Artigas

**Affiliations:** 1 Department of Cell Physiology and Molecular Biophysics and Center for Membrane Protein Research, Texas Tech University Health Sciences Center, Lubbock, TX, USA; 2 Department of Chemistry, Faculty of Science, Hokkaido University, Sapporo, Japan; 3 Department of Biomedicine, Aarhus University, Aarhus, Denmark; 4 Section of Pediatric Neurology, Department of Pediatrics, University of Chicago, Chicago, IL, USA

## Abstract

Various dominant disease phenotypes are caused by germline variants of *ATP1A1*, the gene for the ubiquitously expressed Na^+^,K^+^-ATPase (NKA) α1 subunit. What characteristics link specific variants to a particular disease phenotype remains unclear. We used heterologous expression in mammalian cells and *Xenopus* oocytes to evaluate the characteristics of α1 variants causing hypomagnesemia with treatment-resistant seizures: L302R, G303R, M859R, and W931R. Cell survival in the presence of micromolar ouabain was reduced in cells expressing ouabain-resistant versions of these variants, indicating defective function of the variants relative to WT. Fluorescently tagged WT-α1 expressed in HEK293 cells showed plasmalemma localization, while all variants displayed increased cytosolic signal. In variant-expressing oocytes under voltage clamp, each variant showed passive “leak” currents, which were unaffected by ouabain in L302R and M859R, partially inhibited by ouabain in G303R and enhanced by ouabain in W931R. Na^+^ and Cl^‒^ permeate through G303R and W931R. M859R and W931R presented small K^+^-activated ouabain-inhibitable NKA currents that were absent in L302R and G303R. Growth of cells in ouabain and enzymatic studies demonstrated Na^+^/K^+^-dependent active transport for W931R, albeit with reduced Na^+^ affinity. We solved the cryo-EM structure of W931R-α1β1 with bound ouabain at 3 Å resolution. The structure showed a distorted transmembrane segment M9, which causes a concavity in the detergent micelle, suggesting that similar distortions may provide a leak pathway at the protein–lipid interface. Our results demonstrate that besides a loss of NKA function, all four variants carry an aberrant leak current that probably underlies the seizures and the hypomagnesemia characterizing this phenotype.

## Teaser

Na/K pump α1 subunit variants cause several disease phenotypes. Here, we show that all variants known to cause hypomagnesemia and seizures carry aberrant leak currents and have both reduced function and reduced plasmalemma expression. The cryo-EM structure of variant W931R suggests that the leaking ions may permeate through the protein/lipid interface.

## Introduction

Somatic and germline pathogenic variants of *ATP1A1*, the gene encoding for Na^+^,K^+^-ATPase α1 catalytic subunit, cause numerous diseases. Somatic *ATP1A1* variants in adrenal adenomas are associated with hyperaldosteronism (Azizan et al., 2013; Beuschlein et al., 2013). Germline variants have been associated with Charcot-Marie-Tooth (CMT) neuropathies (Cinarli Yuksel et al., 2023; He et al., 2019; Lassuthova et al., 2018; Spontarelli Fruit et al., 2025) and various more severe phenotypes, including early onset neurodevelopmental syndrome (Chevarin et al., 2020; Dohrn et al., 2022; Lin et al., 2021), complex spastic paraplegia (Stregapede et al., 2020), or hypomagnesemia presenting with treatment-resistant seizures and cognitive delay (Schlingmann et al., 2018; Ygberg et al., 2021).

The Na^+^,K^+^-ATPase (NKA) is an obligatory heterodimer formed by a catalytic α subunit and an auxiliary β subunit but is frequently found as an αβ-FXYD trimer (where FXYD is an NKA regulator protein). There are four different isoforms of the α subunit (coded by *ATP1A1–ATP1A4*), three different isoforms of the β subunit (coded by *ATP1B1–ATP1B3*), and seven FXYD regulators (coded by *FXYD1–FXYD7*). All these isoforms can combine *in vitro* forming isozymes with distinct kinetic characteristics (Blanco, 2005; Crambert et al., 2000; Stanley et al., 2015). However, their physiologic association *in vivo* appears constrained by tissue-specific distribution of each isoform (Blanco, 2005; Geering, 2008). The α1 isoform is ubiquitously expressed. In contrast, α2 is mostly confined to all muscle types and glia, while α3 is neuronal, with a small presence in the heart’s conduction system (but not in all species). Thus, it is logical that *ATP1A2* and *ATP1A3* variants cause exclusively central nervous system (CNS) or neuromuscular pathologies (reviewed by Friedrich et al. [2016]; Holm and Lykke-Hartmann [2016]; Sweadner et al. [2019]). Because of its whole body distribution, pathologic variants in *ATP1A1* cause endocrine, renal, CNS, and peripheral nervous system pathologies (reviewed by Biondo et al. [2021]).

Hypomagnesemia was the first disease found associated with a germline NKA subunit variant. The G41R variant of the FXYD2 protein (also known as γ subunit) was found in multiple members of three Dutch families (de Baaij et al., 2015; Meij et al., 2000). As the FXYD2 protein is almost exclusively expressed in the kidney (Arystarkhova and Sweadner, 2005; Geering, 2006), this renal-only phenotype is accompanied by seizures that respond to magnesium supplementation treatment (Meij et al., 2000). In contrast, the hypomagnesemia reported in patients carrying either of the four α1 subunit variants L302R, G303R, M859R, or W931R is accompanied by cognitive delay and seizures that do not respond to Mg^2+^ supplementation (Schlingmann et al., 2018; Ygberg et al., 2021). This severe Mg^2+^-refractory encephalopathy suggests that the CNS effects are independent of the renal phenotype.

**Figure 1.**
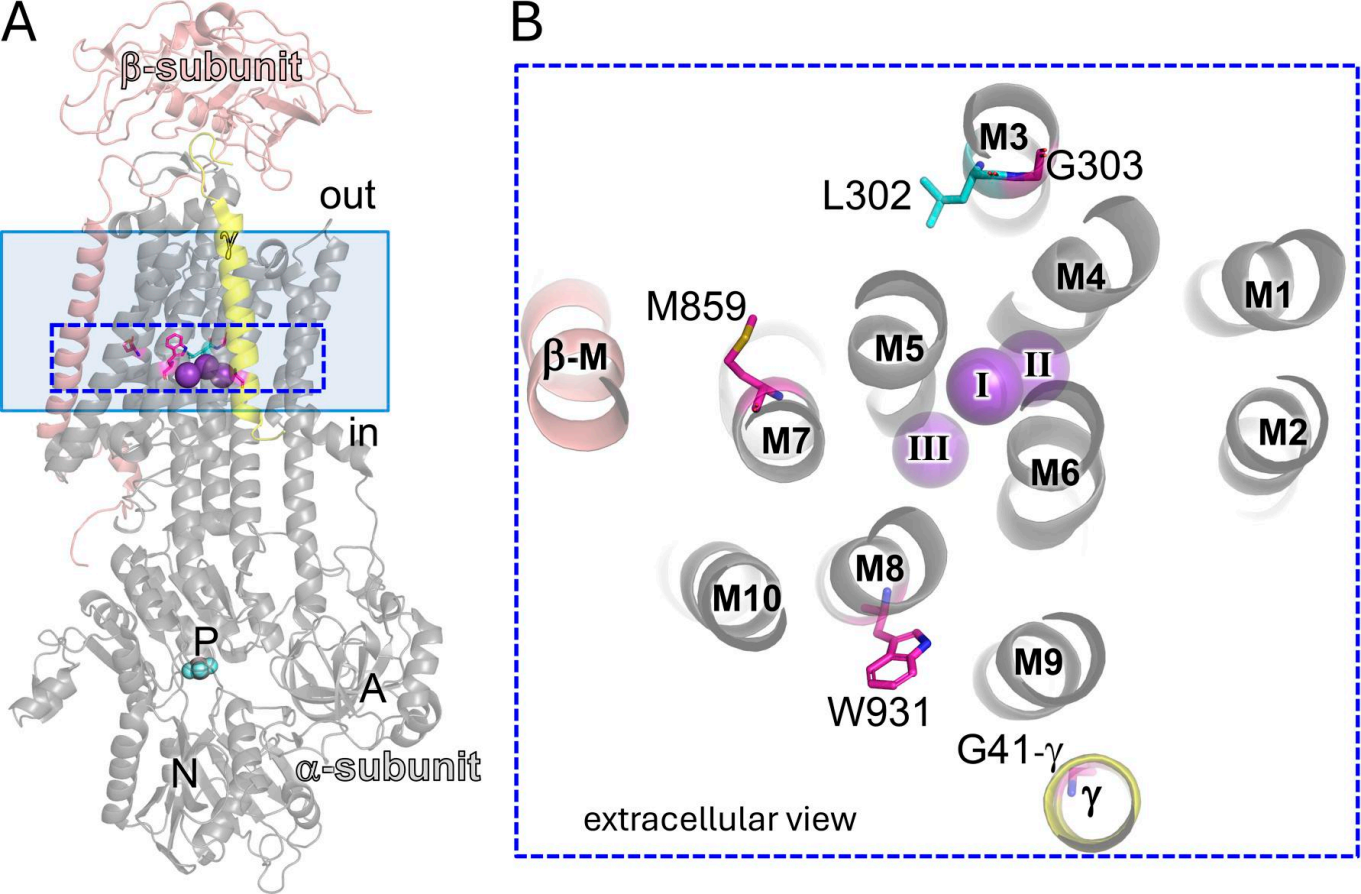
**Location of mutations causing hypomagnesemia in the WT-α1β1g E1(3Na) structure.** Residues examined in this study are shown as sticks (α1 variants L302R, G303R, M859R, and W931R and γ subunit [FXYD2] variant G41R). **(A)** Overall view of the structure from the membrane plane with indication of the three cytoplasmic domains (N, P, and A), with AlF_3_ as phosphate analog (spheres) in the catalytic site of the P domain. **(B)** Extracellular view of the transmembrane region indicated by a dotted box in A. Transmembrane segments M1–M10, β-M, and γ are indicated. Purple spheres are Na^+^ ions indicated by site number. PDB 3WGU (Kanai et al., 2013).

A central question remains: what makes a set of mutations present throughout the body cause a phenotype in one organ system and not the others. One might expect a group of mutations causing a particular phenotype to have disease-specific mechanisms underlying the distinct pathology. Mechanistic studies in various heterologous expression systems have shown that most CMT mutants cause variable degrees of sodium pump loss of function without any evident dominant-negative effects. The four substitutions causing hypomagnesemia with treatment-resistant seizures introduce a large positively charged Arg close to the center of transmembrane segments M3 (L302R and G303R), M7 (M859R), and M8 (W931R). The position of the wild type (WT) residues within the Na^+^-bound structure of the α1β1 NKA is shown in Fig. 1. Reports of the functional characteristics of these *ATP1A1* variants (Schlingmann et al., 2018; Ygberg et al., 2021) show that all four variants have significant loss of function, but the mechanisms for such loss of function, which is also seen in patients with other *ATP1A1-*related phenotypes, remain unclear. On the other hand, L302R, G303R, and W931R (Schlingmann et al., 2018; Ygberg et al., 2021) have been reported to mediate aberrant, passive, inward currents, also called “leak currents,” that could exacerbate the loss of function and provide a phenotype-specific mechanism, particularly for the seizures phenotype. However, such leak current was not observed in M859R.

Here we present a meticulous evaluation of the *ATP1A1* hypomagnesemia variants utilizing functional analysis by electrophysiology and enzymatic assays, expression in HEK293 and COS cells, and cryo-EM structural analysis. Our results uncover common plausible mechanism that could explain development of hypomagnesemia in the nephron and hyperexcitability of the CNS, as all variants presented a drastic loss of function (mediated by reduced expression and reduced interaction with the transported ions) and transported leak currents that could explain the development of the seizure phenotype due to a sustained depolarization of the neurons expressing the disease variants. The novel characteristics we uncovered explain why the common functional features of these variants eluded previous studies, while our cryo-EM structure of the W931R variant suggests a novel mechanism of ion-leak generation where ions could permeate through an alternative pathway in the protein-bilayer interface due to a major distortion of the hydrophobic core of the lipid bilayer.

## Materials and methods

### Ouabain survival assay

The human *ATP1A1* protein coding sequence from Mammalian Gene Collection clone 51,750 (corresponding exactly to GenBank accession no. NM_00701.8) was subcloned into pcDNA3.1+, with two ouabain-resistance mutations Q118R (c.353A>G) and N129D (c.385A>G) introduced by site-directed mutagenesis, which confer ouabain resistance (half-maximal inhibitory concentration [IC_50_] ∼100 µM [Price and Lingrel, 1988]). Additional variants of interest were added by site-directed mutagenesis and verified by whole-plasmid nanopore sequencing (Plasmidsaurus). HEK293 cells were grown in DMEM/F12 supplemented with 10% FBS (Gibco) and 100 µg/ml gentamicin (Thermo Fisher Scientific) at 37°C, 5% CO_2_. Cells were plated into 96-well clear/flat-bottomed plates (Greiner) at 30,000 cells/well, transfected the next day with 120 ng plasmid using a 2.5:1 ratio of lipofectamine 3000 (Thermo Fisher Scientific), and grown for 2 days. Cells were then detached with 30 μl TrypLE Express (Gibco), diluted with 120 μl culture medium, and 50 μl of were transferred to wells containing 100 μl medium with 15 µM ouabain, reaching a final ouabain concentration of 10 μM. Plates without ouabain were prepared in parallel to monitor transfection toxicity. After another 2 days’ growth, cell viability was measured directly in the plate by washing once with 100 μl PBS (Corning) to remove dead cells followed by tetrazolium dye reduction assay (Promega CellTiter Aqueous One Solution). Absorbance at 490 nm in each well was blanked to the average value from 4 wells without cells, then normalized to the average value from wells transfected with the ouabain-resistant (OR) “WT” construct.

### Oocytes experiments

Isolated *Xenopus* oocytes (Xenopus1) were kept in Super Barth Solution (in mM), 88 NaCl, 1 KCl, 0.33 Ca(NO_3_)_2_, 0.41 CaCl_2_, 0.82 MgSO_4_, 2.4 NaHCO_3_, 10 HEPES, 1 Na^+^ Pyruvate supplemented with Gibco anti–anti and 25 μg/ml gentamicin (osmolality ∼170 mOsm/kg, pH 7.4, with NaOH). In some early experiments, the oocytes were kept in SOS solution in which the main difference was the use of 100 mM NaCl and the currently unavailable products horse serum and Gibco Anti–Anti (Spontarelli Fruit et al., 2025).

The cRNA for the human NKA subunits α1 and β1 subunits was *in vitro* transcribed with mMESSAGE mMACHINE SP6 (AM1340; Invitrogen) as described (Spontarelli Fruit et al., 2025). Oocytes were injected with 50 nl of cRNA mixtures containing (by mass) three parts α1 and one part β1 (i.e., equimolar α and β). After injection, oocytes were kept at 16°C until recording. To separate exogenous NKA signals from the oocyte’s endogenous ones, most experiments were performed after introducing the disease variants in an α1 template with the ouabain resistance conferring double substitution Q118R/N129D. This allows inhibiting the endogenous ouabain-sensitive (OS) NKA (IC_50_ <100 nM) by preincubation with 10 μM ouabain. We will refer to this template as the OR WT α1. For some experiments, the disease variants were also evaluated after introduction in the OS WT α1 template, in which case the oocytes were not preincubated with ouabain.

Two-electrode voltage clamp (TEVC) was performed 3–7 days after injection as described (Spontarelli et al., 2022) using an OC-725C amplifier (Warner Instruments) controlled by pClamp software (Molecular Devices). All currents were digitized at 10 kHz with a Digidata 1440A converter and continuously recorded with a Minidigi 1B at 1 kHz (both AD converters from Molecular Devices). Glass electrodes filled with 3 M KCl had 0.2–2 MΩ resistance.

Before recording, oocytes were incubated in Na^+^-loading solution containing (in mM) 90 NaOH, 20 tetraethylammonium-OH, 40 HEPES, and 0.2 EGTA, osmolality ∼220 mOsm/kg, pH 7.2, with either sulfamic acid or methanesulfonic acid (MS), and supplemented with 10 μM ouabain for oocytes injected only with OR NKA. After loading, oocytes were kept in 125 mM Na^+^ recording solution (osmolality ∼260 mOsm/kg) containing (in mM) 125 NaOH, 5 Ba(OH)_2_, 1 Mg(OH)_2_, 0.5 Ca(OH)_2_, and 10 HEPES, pH titrated to 7.4 or 7.6, with MS. Other recording solutions are named after their main extracellular cation where 125 mM N-Methyl D-glucamine (NMDG), 125 mM KOH, or 150 mM NaOH substitute for 125 mM NaOH (osmolality ∼300 mOsm/kg). In addition, to test for Cl^−^ permeability, we used a 125-mM NMDG solution in which MS was substituted by Cl^−^ (NMDG-Cl solution). The use of 7.4 or 7.6 pH did not affect the currents at −60 mV, even in the absence of Na^+^ and K^+^, where protons leak through the WT NKA (Mitchell et al., 2014). Solutions that contained Na^+^ and K^+^ were made by mixing the Na^+^ solution with a K^+^ solution (125 or 150 mM). Ouabain was directly dissolved in external solutions.

Electrophysiological data were analyzed using pClamp (Molecular Devices). The mean steady-state current was measured during the last 5 ms of the voltage pulses and plotted against the applied voltage. I–V curves were then subtracted to obtain the difference-currents plotted. OriginLab was used for statistical analysis.

### Enzymatic measurements on mutant W931R expressed in COS-1 cells

The W931R mutation was introduced by PCR into cDNA encoding the naturally OR rat α1-isoform of NKA. Variant and WT cDNAs were transfected into COS-1 cells using the Ca^2+^-phosphate precipitation method (Chen and Okayama, 1987), and the cells were grown under ouabain selection pressure (Jewell-Motz and Lingrel, 1993; Vilsen, 1992). Because ouabain inhibits the endogenous, OS COS-1 cell NKA, cell survival, and further growth depend on the ability of the exogenous NKA to become expressed in the plasma membrane (PM) and transport Na^+^ and K^+^. The presence of the W931R mutation was verified by full-length cDNA sequencing before transfection as well as following isolation of stable transfectants, where it had become integrated in the genome.

Crude PM vesicles from the isolated cell lines containing either the expressed W931R mutant or WT NKA were prepared by differential centrifugation. For functional analysis, the membranes were made leaky using alamethicin to allow access of ATP, Mg^2+^, and the transported Na^+^ and K^+^ ions from both sides (Nielsen et al., 2024). The maximum NKA activity was determined in the presence of saturating Na^+^ and K^+^ concentrations at 37°C by following the liberation of P_i_ (Baginski et al., 1967) in medium containing 30 mM histidine (pH 7.4), 130 mM NaCl, 20 mM KCl, 1 mM EGTA, 3 mM MgCl_2_, 3 mM ATP, and 10 μM ouabain (inhibiting the endogenous COS-1 cell NKA). To determine the K^+^ dependence of NKA activity, the same protocol was used except that the medium contained 40 mM NaCl and varying concentrations of KCl. Na^+^-ATPase activity was determined at varying concentrations of NaCl in the absence of K^+^. In all cases, the background activity obtained from similar measurements in the presence of 10 mM ouabain (with all NKAs inhibited) was subtracted.

To determine the maximum capacity for phosphorylation from [γ-^32^P]ATP (active site concentration), the leaky membranes were incubated at 0°C in the presence of 2 μM [γ-^32^P]ATP in a medium containing 20 mM Tris, pH 7.5, 150 mM NaCl, 3 mM MgCl_2_, 1 mM EGTA, 20 μg oligomycin/ml to prevent dephosphorylation, and 10 μM ouabain. For Na^+^ dependence of phosphorylation, various concentrations of NaCl were present together with a varying concentration of NMDG to maintain the ionic strength. For background subtraction, the phosphorylation experiments were performed in the presence of 50 mM KCl without added NaCl under otherwise the same conditions as above. The formation of phosphoenzyme was terminated after 10-s incubation with [γ-^32^P]ATP by quenching with ice-cold 1 M phosphoric acid. The acid-precipitated ^32^P-labeled phosphoenzyme was washed using centrifugation prior to SDS-PAGE at pH 6.0. The radioactivity associated with the separated ^32^P-labeled phosphoenzyme band was quantified by phosphor imaging using a Cyclone Plus Storage System (Model C431200; PerkinElmer).

The Na^+^ and K^+^ dependences were analyzed by nonlinear regression using the SigmaPlot program (SPSS, Inc.), and the best fits are shown as lines in the figures. The Hill equation for binding of an activating ligand (*L*) was applied

$$A=\left(A_{\max}-A_{0}\right)\cdot \frac{{\left[L\right]}^{n}}{{\left(K_{0.5}\right)}^{n}+{\left[L\right]}^{n}}+A_{0}$$

*A*_*max*_ is the maximum level of ATPase activity or phosphorylation corresponding to infinite ligand concentration, *A*_0_ defines the value in the absence of ligand (only of use for *L* = K^+^ where some activity is seen in the absence of ligand).

### Protein expression, purification, and cryo-EM analysis

The procedures for protein expression are essentially as reported (Abe et al., 2018; Nakanishi et al., 2020). Briefly, an octa-histidine tag and the enhanced GFP (EGFP) were inserted in the amino terminal side of N-terminal 38 amino acid of the human NKA α1-subunit (WT or W931R variant), followed by the tobacco etch virus (TEV) protease recognition sequence and subcloned into a handmade pFBM vector. The WT human NKA β1-subunit was also cloned. The αβ complex of NKA were expressed using baculovirus-mediated transduction of mammalian human embryonic kidney (HEK293S GnT1^–^) cells (Dukkipati et al., 2008), purchased from ATCC.

For cryo-EM analysis, cells were directly solubilized with 1% lauryl maltose neopentyl glycol (Chae et al., 2010) in the presence of 40 mM MES/Tris (pH 6.5), 10% glycerol, 5 mM dithiothreitol, 50 mM NaCl, and 1 mM MgCl_2_, in the presence of 1 mM BeSO_4_, 3 mM NaF, 0.1 mM ouabain, and protease inhibitor cocktail (Roche) on ice for 20 min. After removing insoluble material by ultracentrifugation, the supernatant was mixed with anti-GFP nanobody resin (Kubala et al., 2010) at 4°C for 2 h, which was followed by washing with buffer containing 40 mM MES/Tris (pH 6.5), 2% glycerol, 1 mM MgCl_2_, 1 mM BeSO_4_, 3 mM NaF, 50 mM NaCl, 0.05 mM ouabain, and 0.06% glyco-diosgenin (GDN) (Chae et al., 2012). After addition of TEV protease and endoglycosidase, anti-GFP nanobody was incubated at 4°C overnight. Digested peptide fragments containing EGFP and endoglycosidase were removed by passing the fractions through Ni-NTA resin (Qiagen). Flow-through fractions were concentrated and subjected to size-exclusion column chromatography using a Superose6 increase column (Cytiva) equilibrated in buffer comprising 20 mM MES/Tris (pH 6.5), 1 mM MgCl_2_, 1 mM BeSO_4_, 3 mM NaF, 50 mM NaCl, 0.05 mM ouabain, and 0.06% GDN. Peak fractions were collected and concentrated to 8 mg/ml, and 0.1 mM ouabain was added to the protein sample, which was prepared for cryo-EM grids as previously reported (Young et al., 2022). For the K^+^-occluded form, 1 mM BeSO_4_, 3 mM NaF, 50 mM NaCl, and 0.05 mM ouabain were replaced by 1 mM AlCl_3_, 4 mM NaF, and 200 mM KCl in the solution. Purified protein samples were applied to a freshly glow-discharged Quantifoil holey carbon grids (R1.2/1.3, Cu/Rh, 200 mesh), using a Vitrobot Mark IV (Thermo Fisher Scientific) at 4°C with a blotting time of 4 s under 99% humidity, and then plunge-frozen in liquid ethane. The prepared grids were transferred to a CRYO ARM 300 microscope (JEOL), operated at 300 kV, with a cold-field emission gun as the electron source, an in-column Omega filter, and equipped with a Gatan K3 direct electron detector in the electron counting mode. Imaging was performed at a nominal magnification of 60,000×, corresponding to a calibrated pixel size of 0.753 Å/pix (EM01CT at SPring-8). Each movie was recorded in correlated-double sampling electron counting mode for 2.6 s and subdivided into 60 frames. The electron flux was set to 8.46 e^−^/pix/s at the detector, resulting in an accumulated exposure of 60 e^−^/Å^2^ at the specimen. The data were automatically acquired by the image shift method using SerialEM software (Mastronarde, 2005), with a defocus range of −0.8 to −1.8 μm. The dose-fractionated movies were subjected to beam-induced motion correction, using RELION 3.1 (Zivanov et al., 2018), and the contrast transfer function (CTF) parameters were estimated using patch CTF estimation in cryoSPARC (v4, Structura Biotechnology) (Punjani et al., 2020).

For each dataset, particles were initially picked by blob picker using cryoSPARC (v4) and extracted with down-sampling to a pixel size of 3.24 Å/pix. These particles were subjected to several rounds of 2D classifications. Selected classes were then subjected to *ab initio* reconstruction in three models and refined by nonuniform refinement (Punjani et al., 2020). The particles from the best class were then re-extracted to the full pixel size and subjected to nonuniform refinement with per-particle defocus refinement and beam-tilt refinement in cryoSPARC (v4). The particle stack was then transferred to RELION 3.1 and subjected to Bayesian polishing (Zivanov et al., 2019). Polished particles were reimported to cryoSPARC (v4) and subjected to nonuniform refinement. The resolution of the analyzed map was defined according to the fourier shell correlation (FSC) = 0.143 criterion (Rosenthal and Henderson, 2003) (Table S1). The local resolution and angular distributions for each structure were estimated by cryoSPARC (v4). All the models were manually built in Coot (Emsley and Cowtan, 2004) using the homology model derived from a crystal structure of ouabain-bound NKA as a template (Protein Data Bank [PDB] 7WYT, Kanai et al., 2022). Refinement was done with Phenix version 20 (Adams et al., 2010).

### Imaging of transfected fluorescent α1

The procedures for cell culture, transfection, and imaging were essentially as described (Spontarelli Fruit et al., 2025; Spontarelli et al., 2024). HEK 293 cells (Passage 8) were maintained in DMEM supplemented with 10% FBS and 2% penicillin-streptomycin at 37°C in a humidified atmosphere with 5% CO_2_, plated in 6-well plates (2.5 × 10^5^ cells/well), and cultured until reaching 70–80% confluency, when they were transfected using PolyJet In Vitro DNA Transfection Reagent (SL100688; SignaGen Laboratories), following the manufacturer’s instructions. Three plasmids (a gift from Seth Robia at Loyola University, Chicago, IL, USA) were co-transfected in a one to one mass ratio: two containing the cDNA for the human NKA subunits α1 and one containing β1. The coding region for the OR α1 was preceded by the coding sequences of either the YFP or the mCerulean fluorescent protein (CFP). Fluorescent proteins connected to the α1 N terminus with a 10-residue linker. NKAs with this N-terminally tagged α1 are functional (Bossuyt et al., 2009). The disease variants were introduced in the YFP clone by mutagenesis and confirmed by full-plasmid sequencing.

The PolyJet reagent was removed from the culture media 12 h after transfection. A day later, the cells were detached using 0.25% trypsin-EDTA and subsequently plated onto poly-L-lysine–coated coverslips where they were allowed to adhere for 6 h (to ensure a more-or-less circular cell morphology). The coverslips were fixed with 4% paraformaldehyde (in PBS), mounted using ProLong Diamond Antifade Mountant (P36965; Invitrogen) and hardened for a day at 4°C before imaging. Images were acquired with a 40× oil-immersion objective on a Nikon T1-E confocal microscope. Excitation laser wavelengths were 445 nm for CFP and 514 nm for YFP. The images were acquired at the emission wavelengths of 485 ± 35 and 545 ± 30 nm, respectively. Images used for cellular localization analysis were captured with a resolution of 512 × 512 pixels to minimize fluorescent probe bleaching. Higher-resolution images selected for display were acquired at 1,024 × 1,024 pixels.

Relative PM localization of fluorescent subunits was quantified in isolated cells with Fiji image analysis software (Schindelin et al., 2012). The two manually defined regions of interest (ROIs) were the external cell perimeter and the cytosolic perimeter. The PM area and fluorescence intensities were determined by subtracting the cytosolic ROI from the external cell perimeter ROI. The total intensity of each ROI was normalized by its area, and the PM/cytosolic intensity-ratio was calculated. Identical ROIs were applied to YFP and CFP channels.

### Online supplemental material

**Figure S1.**
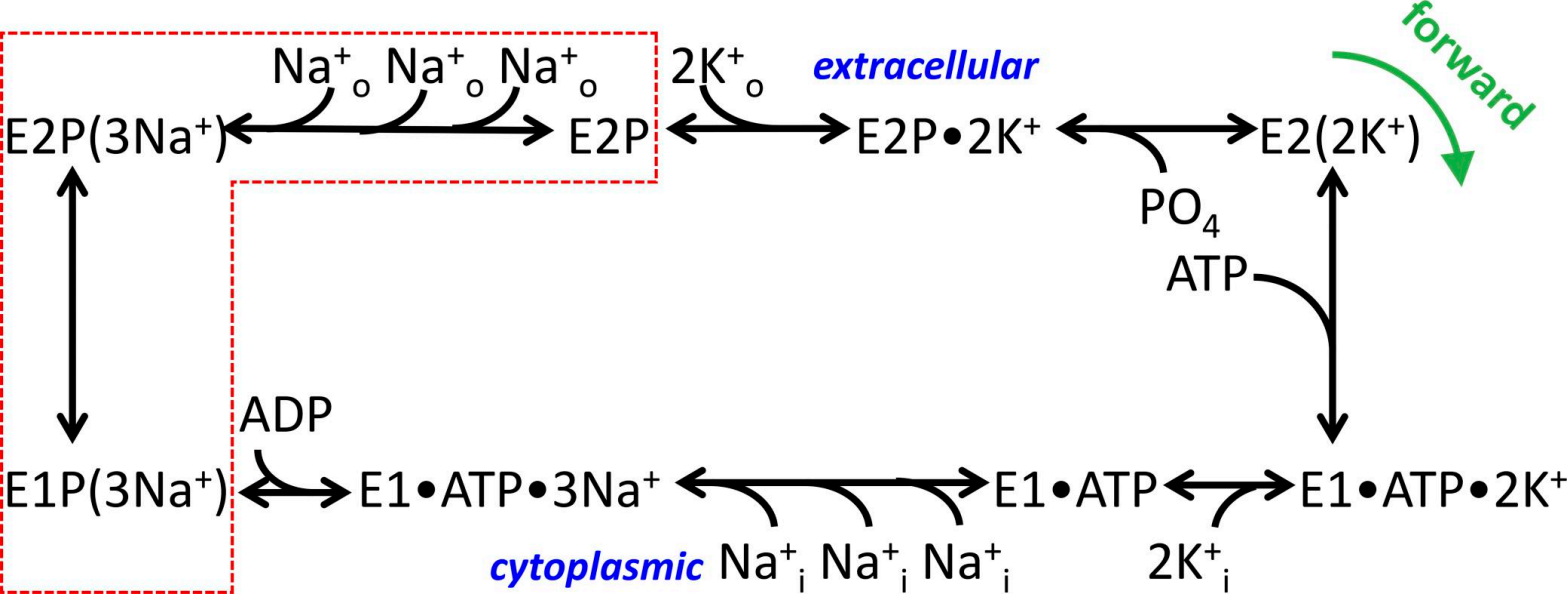
**Post-Albers kinetic scheme describing NKA function.** The figure is reproduced from Artigas et al. (2023). Through its catalytic cycle, the NKA transits through phosphorylated and dephosphorylated forms of two major conformations: E1, bottom row, and E2, top row (Post et al., 1972). As the pump extrudes one charge per cycle, it produces an outward current (seen when K^+^ is added to move the cycle forward in the main figures of the manuscript (Figs. 3, 4, 5, 6, 7, 8, and 9). The red dotted box corresponds to the states in the cycle that undergo transitions that depend on the external [Na^+^] and the voltage across the membrane, producing the transient currents observed in Fig. 3 C. External Na^+^ rebinding to the Na^+^-specific site-III is responsible for the inhibition phase of the Na^+^-ATPase activity of Fig. 10 E, this is the same backward reaction that is favored by voltage in the presence of Na^+^ in Fig. 3 C. The activation phase of the Na^+^-ATPase activity (where Na^+^ replaces K^+^ in the forward reaction cannot be distinguished in electrophysiology experiment because it is more than 100-times slower than the Na^+^- and K^+^-dependent activity (compare with Na^+^, K^+^ turnover in Fig. 10 B) and is not indicated in the kinetic scheme. In the electrophysiological experiments, ouabain binds preferentially to the E2P state with two Na^+^ ions bound (Stürmer and Apell, 1992).

**Figure S2.**
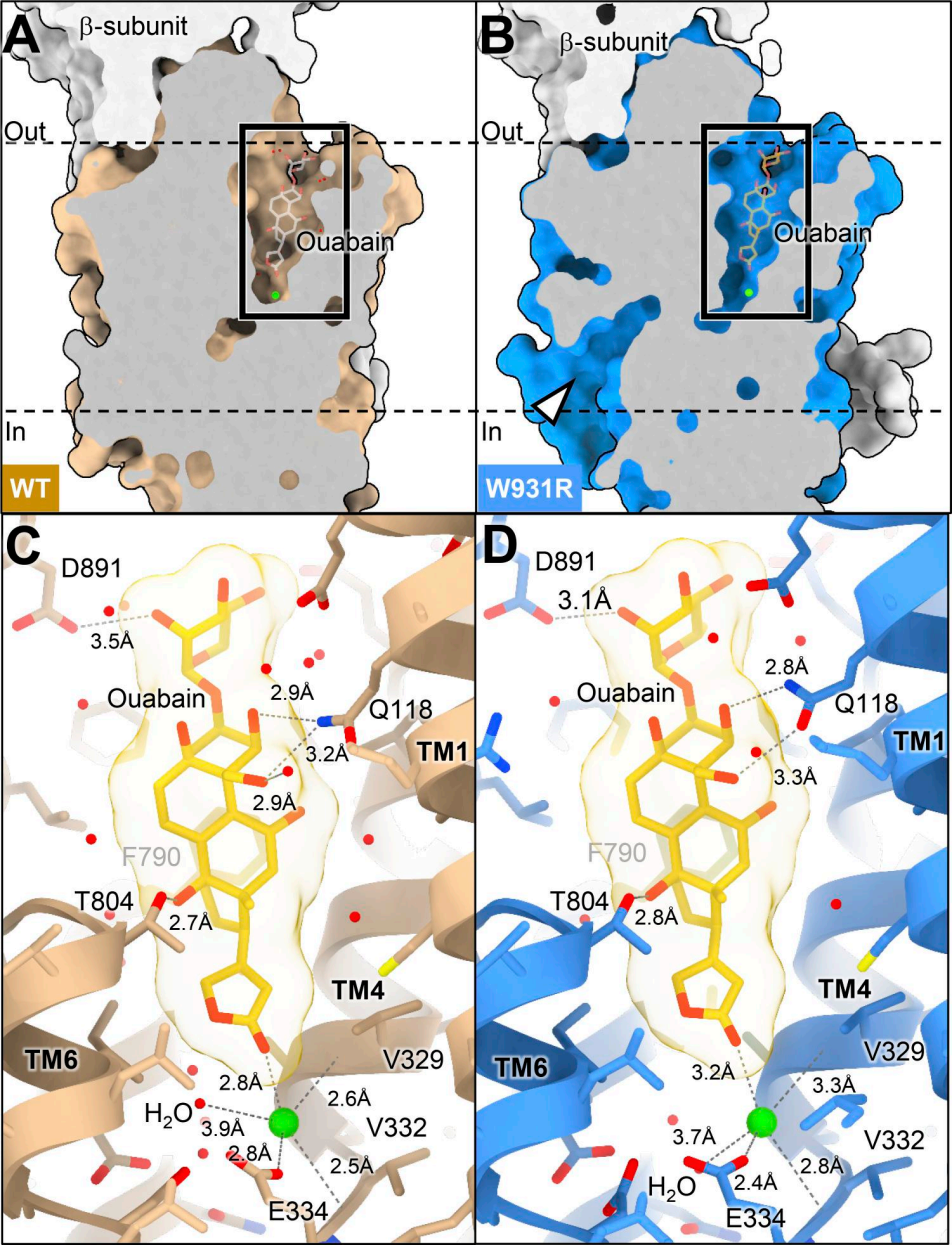
**Ouabain-binding pocket. (A and B)** Surface representation of clipped membrane slices of WT (A) and W931R (B). Dotted lines indicate the approximate location of the membrane plane. A white arrowhead indicates cytoplasmic exposure due to M9 disorder. **(C and D)** Zoomed-in view of the boxed region in A and B showing the molecular details of ouabain (yellow carbons) and Mg^2+^ (green spheres) interactions for WT (C) and W931R (D). Water molecules are displayed as red spheres.

**Figure S3.**
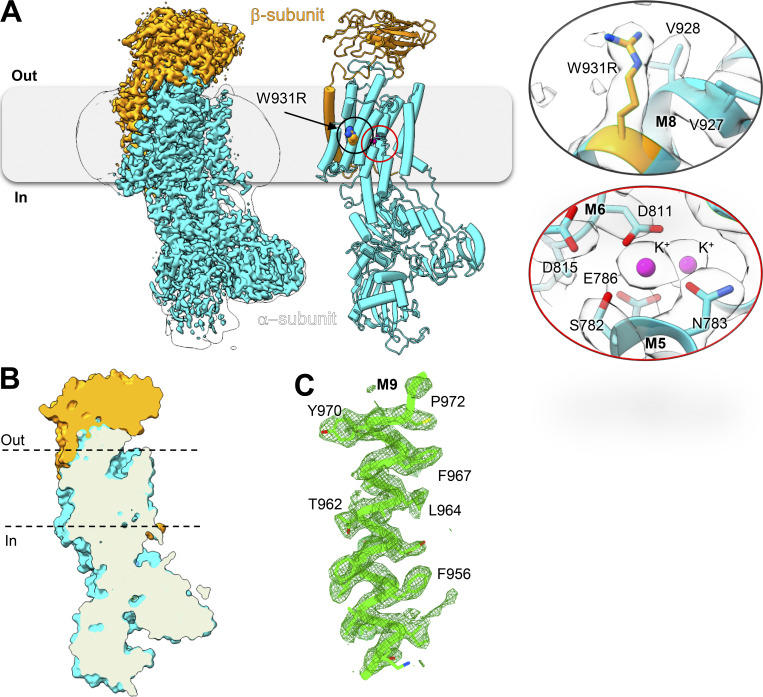
**Cryo-EM structure of W931R variant in the K** ^**+**^ **-occluded E2-P**_**i**_ **state. (A)** Left, side view of the EM density map. Middle, cartoon model with Arg931 shown in yellow carbons with blue nitrogen atoms, with the two occluded K^+^ ions shown as purple spheres. Right, close-up views of W931R and the K^+^-binding sites. **(B)** Surface representation of clipped membrane slices of W931R 2K^+^E2-P_i_ form. Dotted lines indicate approximate location of the membrane plane. **(C)** Sharpened EM density map of M9 superimposed with its cartoon model.

The three supplementary figures provide the Post-Albers scheme describing Na, K-ATPase function (Fig. S1), structural details of the binding of ouabain to WT and W931R NKAs (Fig. S2), and highlights of cryo-EM structure of the W931R mutant with occluded K^+^ (Fig. S3). Table S1 provides parameters of the structural models.

## Results

### Decreased survival of cells expressing variant ATP1A1

**Figure 2.**
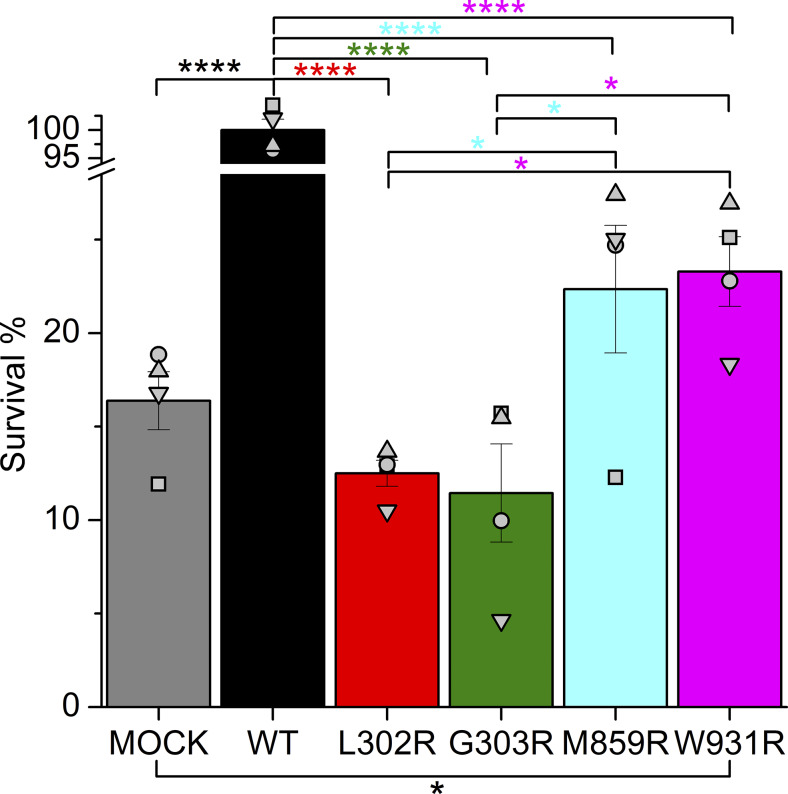
**Ouabain survival of OR-α1 variants.** Mean survival of HEK293 cells in 10 μM ouabain after transfection with different OR-ATP1A 1 variants (bar graphs) overlapped with individual data, normalized to survival of cells transfected with WT ATP1A1. Error bars show SEM. Note the break on y axis. The horizontal segments indicate significance of *t* test comparing the means at P < 0.05 (*) and P < 0.0001 (****) levels.

To verify and compare the deleterious effects of the four variants associated with hypomagnesemia and epilepsy, we first evaluated whether they affect cell survival in the ouabain-complementation assay (Fig. 2). Ouabain is a specific inhibitor of the NKA. Since NKA activity is essential for cell survival, exposing cells to 10 μM ouabain, a concentration that inhibits 100% of endogenous NKA, kills the cells. If cells are transfected with exogenous functional NKAs that have reduced affinity for ouabain (OR NKAs in which ouabain acts with much lower affinity, IC_50_ ∼100 μM, see Materials and methods) cell survival in 10 μM ouabain increases. Comparison of the survival in ouabain upon transfection of an OR disease variant to the survival upon transfection of OR WT is commonly used as a general readout of whether genetic variants have deleterious effects. Consistent with prior reports showing significant loss of NKA function, all four variants significantly decreased cell survival in HEK293 cells relative to the ATP1A1 WT (*t* test, P < 0.0001), while cells with M859R or W931R had significantly greater survival than cells with L302R, G303R, or mock-transfected cells.

### Electrophysiological characteristics of ATP1A1 variants

**Figure 3.**
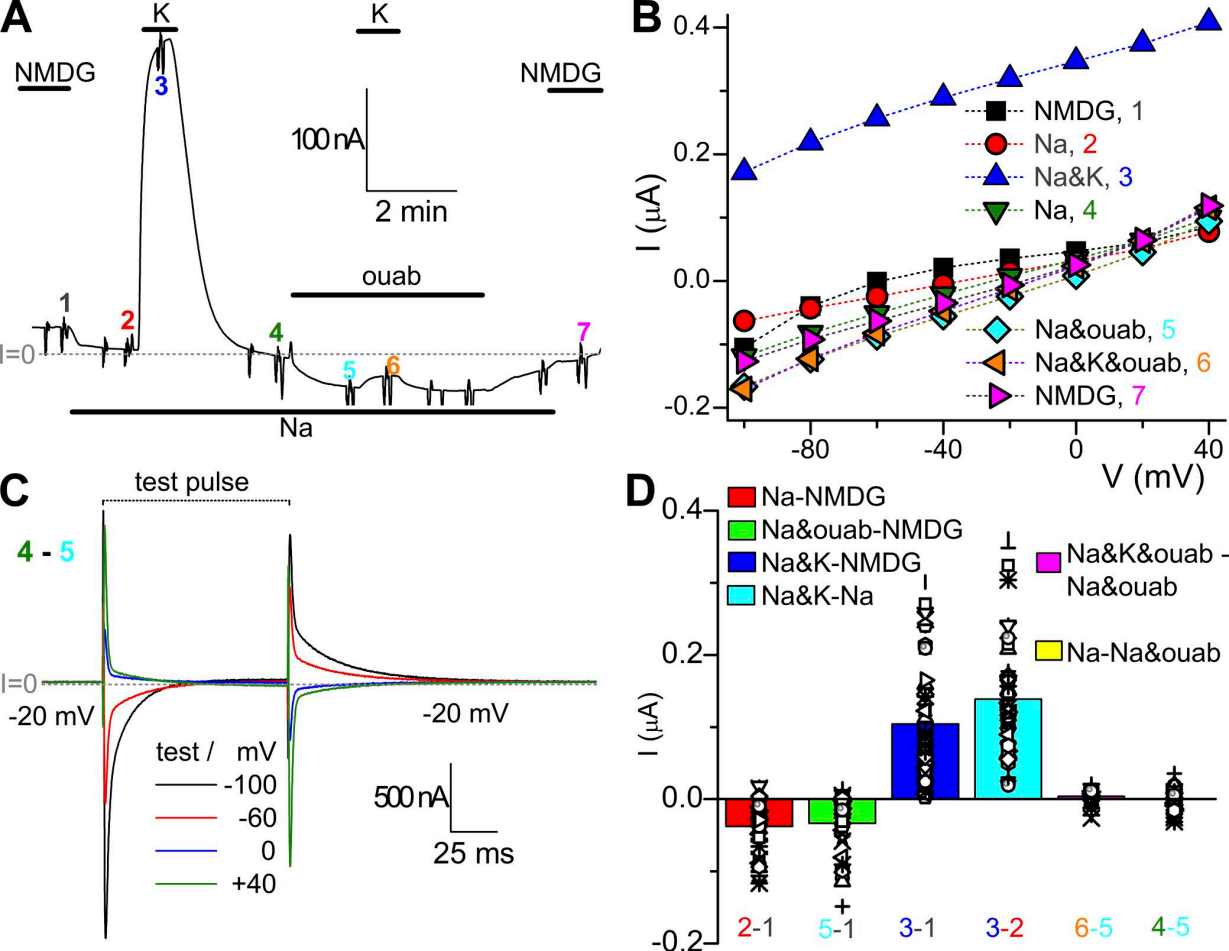
**Electrophysiology of OR-WT α1β1. (A)** Representative oocyte injected with WT-α1β1, held at −20 mV, and bathed in NMDG solution. Switching to 150 mM Na^+^ had minimal effect on the holding current. Application of 4.5 mM K^+^ reversibly activated outward NKA current. Subsequent exposure to 10 mM ouabain in Na^+^ solution slightly altered the holding current and prevented subsequent activation of NKA by K^+^. The small remnant current appears unspecific, probably reflecting relief of Ba^2+^ blocking of K^+^ channels at −20 mV (Armstrong et al., 1982). After ouabain withdrawal the current promptly returned to baseline, indicating that this small effect of the inhibitor is not through the NKA, which requires 10 min to fully recover. Return to NMDG at the end of the experiments illustrates oocyte stability throughout the experiment, despite a small time-dependent increase in unspecific leak evident when comparing with the dotted zero current line. Vertical deflections represent application of 100-ms-long square voltage pulses to measure steady current voltage (I–V) curves. **(B)** I–V curve plotting the average current during the last 5 ms of the pulse vs. the applied voltage at the times indicated by numbers in A. **(C)** NKA-mediated transient current elicited by pulses to the indicated voltage; current before ouabain – current in 10 mM ouabain (subtraction of 4–5 in the compressed timescale trace in A). **(D)** Difference-current between various extracellular solutions at −60 mV, with the mean shown as bar graphs overlapped with the symbols for all individual experiments. The large amplitude variability reflects variable expression levels. For each disease variant, part of this dataset conform the WT control bar plots shown in subsequent figures.

We used TEVC electrophysiology to compare the functional characteristics of the NKAs formed by the OR α1 subunit variants associated with β1 expressed in *Xenopus* oocytes. We focused on effects at physiological extracellular ion concentrations in humans (i.e., 145.5 mM Na^+^ and 4.5 mM K^+^) with near saturating intracellular Na^+^ (Materials and methods). First, we describe the experiments in Na^+^-loaded oocytes injected with OR-WT α1β1 to illustrate the measurements performed with all variants (Fig. 3). Fig. 3 A shows a trace from an oocyte at −20 mV in which after a brief period in an external solution containing NMDG, the perfusate was switched to a solution with only 150 mM Na^+^, causing minor effects on the holding current. Application of 4.5 mM K^+^ reversibly activated a large outward NKA current (due to the electrogenic transport of 3 Na^+^ in exchange for two K^+^ in each NKA cycle [Fig. S1]). Subsequent perfusion of 10 mM ouabain in Na^+^ solution caused a small dip in current and then prevented activation of NKA by a second K^+^ application. The vertical deflections seen indicated by numbers throughout the trace correspond to 100-ms-long pulses from −120 to +40 mV applied to measure the steady-state current as a function of voltage (Fig. 3 B). Ouabain also inhibits the Na^+^- and voltage-dependent conformational changes that the NKA undergoes in the absence of K^+^ (dotted box on the Post-Albers kinetic scheme in Fig. S1). These conformational transitions normally produce ouabain-inhibited transient currents induced by square voltage pulses (Fig. 3 C). The panel illustrates that the large transient currents are not accompanied by ouabain-inhibitable steady-state current in WT expressing oocytes.

For simplicity, we only illustrate the analysis of the difference between the currents measured at −60 mV under several conditions (Fig. 3 D). The bar graphs show the average for (1) current in Na^+^ minus current in NMDG (red), (2) current in Na^+^ with ouabain minus current in NMDG (green), (3) current in Na^+^ and K^+^ minus current in NMDG (blue), (4) current in Na^+^ and K^+^ minus current in Na^+^ alone (cyan, K^+^-induced in Na^+^), (5) current in Na^+^, K^+^ and ouabain minus the current in Na^+^ and ouabain (magenta, K^+^-induced current in ouabain), and (6) current in Na^+^ minus the current in Na^+^ and ouabain (yellow). Overlapped data points from individual oocytes are identified by the same symbol across the different conditions.

**Figure 4.**
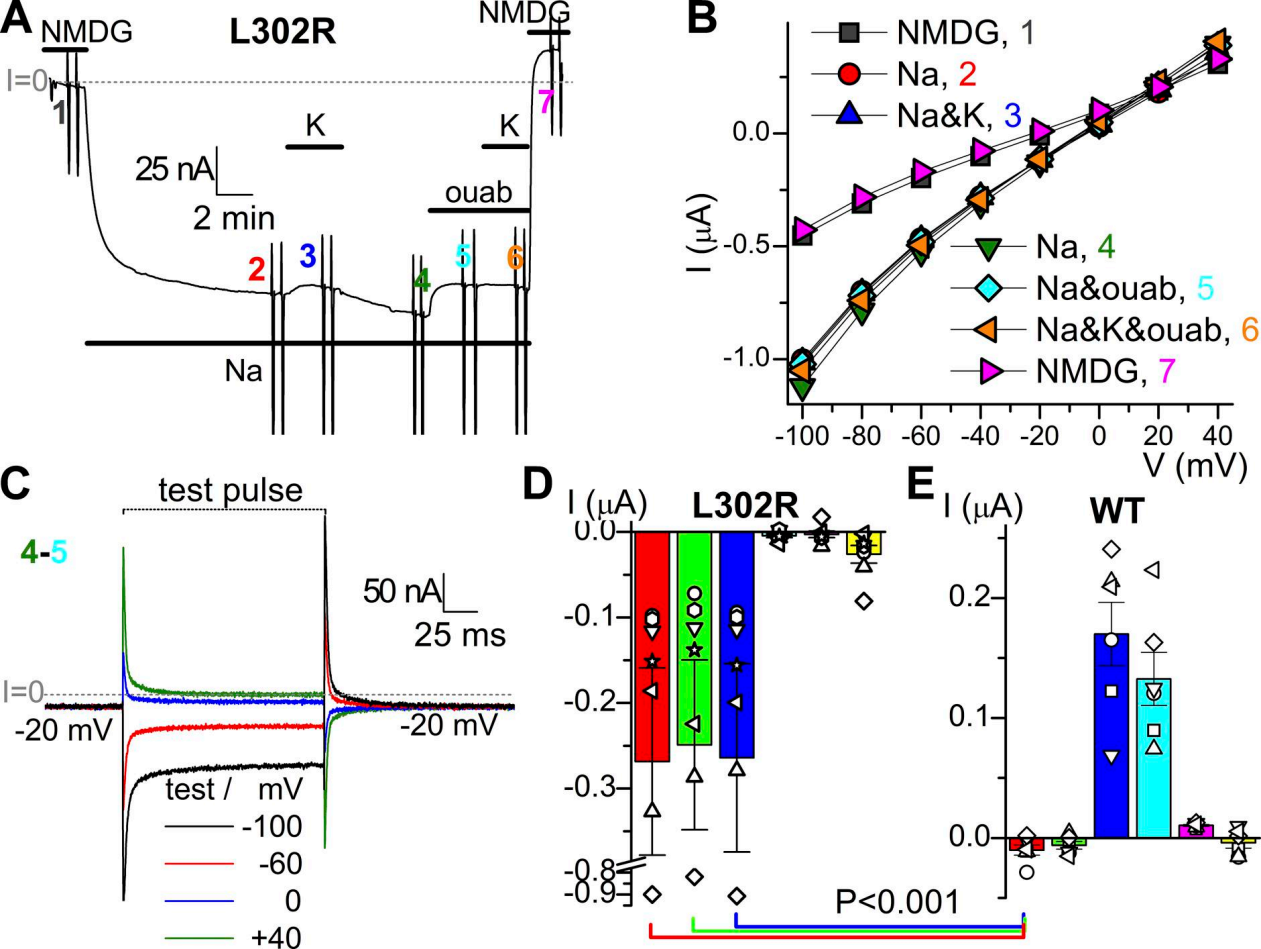
**Electrophysiological characteristics of the OR-L302Rα1β1. (A)** An oocyte injected with OR-L302R was held at −20 mV. A large inward current was observed when switching from NMDG to 150 mM Na^+^ solution. Application of 4.5 mM K^+^ failed to activate significant outward current. Ouabain (10 mM) caused a small outward shift on the holding current. Return to NMDG demonstrates oocyte stability throughout the experiment. **(B)** I–V curves obtained in different external solutions at the times indicated in A. Note that only a fraction of the inward current induced by the switch to Na^+^ is inhibited by ouabain. **(C)** Ouabain-inhibited current elicited by pulses at the indicated voltages. **(D)** Mean difference-currents at −60 mV overlapped with all individual experiments as open symbols. **(E)** Bar graph with the equivalent subtractions in control WT-injected oocytes measured on the same dates as those in D. Horizontal lines indicate the mean difference-currents with significantly larger inward current than the Na^+^ minus NMDG subtraction, the largest inward current from WT oocytes (*t* test). Bar color code as in Fig. 3 D.

An identical protocol was used to evaluate the four *ATP1A1* variants. Oocytes expressing the M3 substitution OR-L302R α1 display obvious differences with those injected with the OR-WT α1 (Fig. 4). The results from a representative oocyte (Fig. 4, A−C) highlight that (1) switching from NMDG to Na^+^ caused a large inward deflection of the holding current at all voltages (Fig. 4, A and B) and perfusion of 4.5 mM K^+^-activated negligible outward current (Fig. 4, A and B) and 2) application of ouabain caused a small outward deflection of the steady current (Fig. 4 B). The ouabain-inhibited traces in Fig. 4 C show reduced transient current and increased steady-state current at negative voltages. However, these ouabain-inhibited steady-state currents are tiny compared with the Na^+^-induced ones (Fig. 4, B and D). The mean difference-currents at −60 mV show that OR-L302R oocytes demonstrate significantly larger inward currents in all Na^+^-containing solutions (Fig. 4 D red, green, and blue bars) than the Na^+^-induced current in WT oocytes on the same day (Fig. 4 E, red bar).

**Figure 5.**
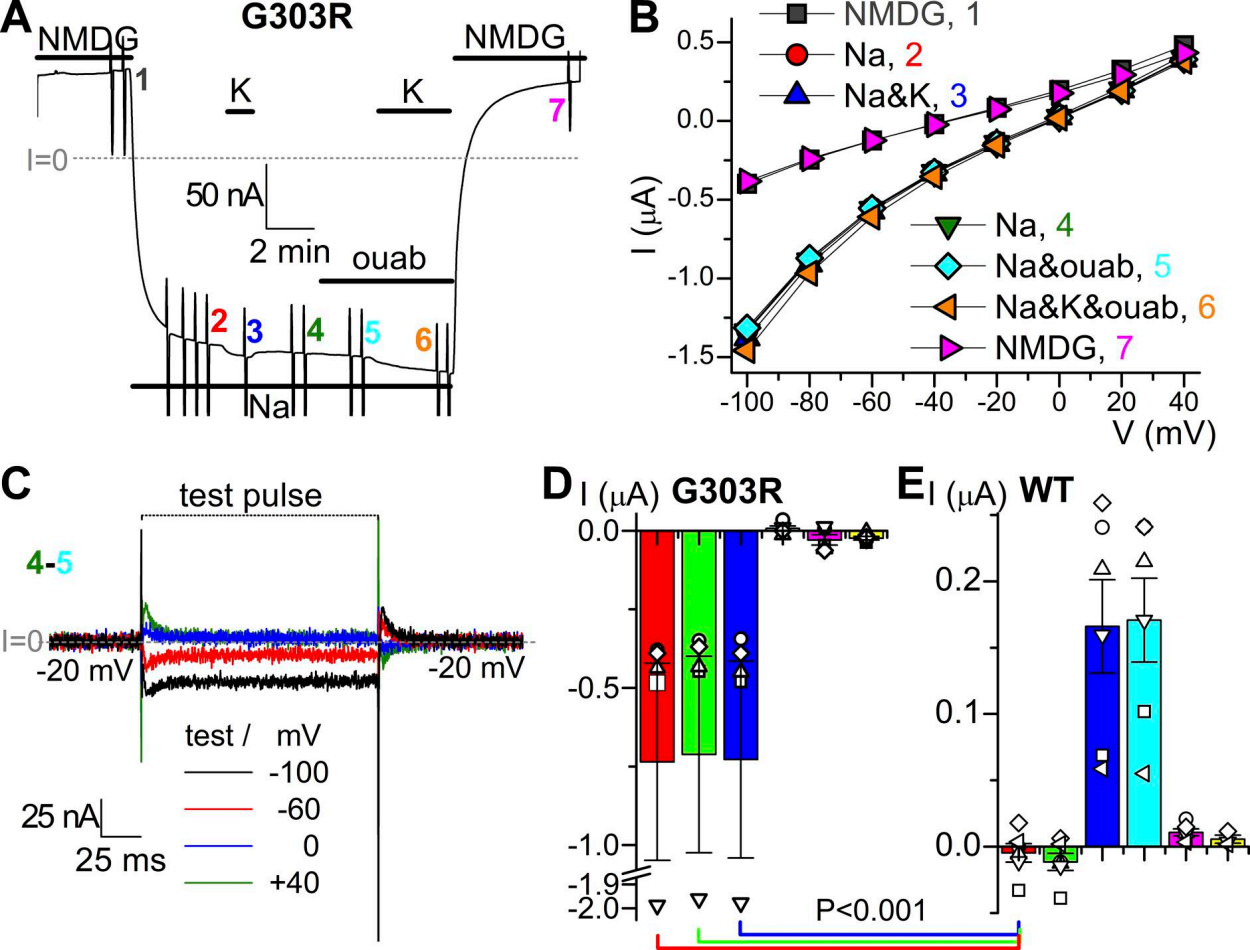
**Electrophysiological characteristics of OR-G303Rα1β1. (A)** An oocyte injected with OR-G303R held at −20 mV showed a large inward current when switching from NMDG to 150 mM Na^+^ solution. Application of 4.5 mM K^+^ failed to activate current. 10 mM ouabain had minimal effects on the holding current. Return to NMDG shows that the oocyte remained stable throughout the experiment. **(B)** I–V curve plotting the average current during the last 5 ms of the pulse vs. the applied voltage at the times indicated by numbers in A. **(C)** Ouabain inhibited current is minimal at all voltages. **(D)** Mean difference-currents at −60 mV overlapped with all individual experiments as open symbols. Note the large inward current when subtracting Na^+^ with K^+^ minus NMDG (blue bar). **(E)** Bar graph with the equivalent subtractions in control WT-injected oocytes measured in the same dates as those in D. Horizontal lines indicate the mean difference-currents with significantly larger inward current than the Na^+^-NMDG subtraction, the largest inward current from WT oocytes (*t* test). Bar graph color code as in Fig. 3 D.

The results with oocytes injected with OR-G303R α1 (Fig. 5) are similar to those with OR-L302R. The main differences are: a larger inward current induced by the switch to Na^+^ solution and the nearly complete insensitivity to ouabain application (Fig. 4 C and Fig. 5 A). The difference-current between any Na^+^ containing solution and NMDG (Fig. 5 D, red, green, and blue bars) are significantly larger than the Na^+^-induced current in WT injected oocytes on the same day (Fig. 5 E, red bar).

**Figure 6.**
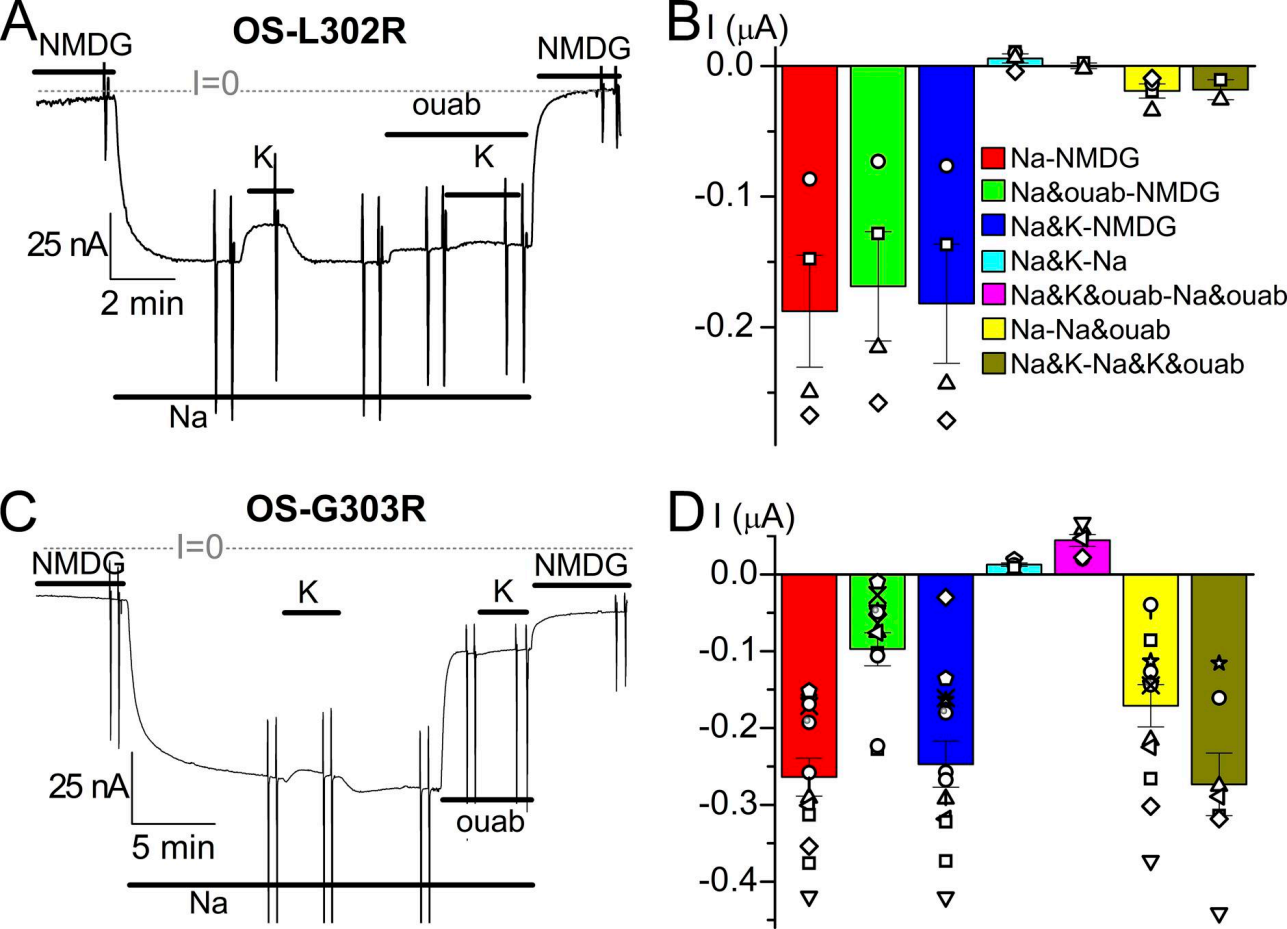
**Currents through OS-L302R-α1 and OS-G303R-α1β1. (A)** Switching from NMDG to Na^+^ solution in an oocyte injected with OS-L302R-α1β1 at −20 mV induced inward current. K^+^ (4.5 mM) activated endogenous NKA current. 1 mM ouabain had a minor effect on holding current and inhibited endogenous NKA activation by subsequent K^+^ application. Return to NMDG shows oocyte stability throughout the experiment. **(B)** Mean difference-current at −60 mV overlapped with symbols for individual experiments. **(C)** Recording from an oocyte-expressing OS-G303R-α1β1 held at −20 mV. Switching to Na^+^ activated inward current. K^+^-activated endogenous NKA. Ouabain inhibited ∼60% of the inward current. **(D)** Mean difference-current at −60 mV overlapped with individual experiments as open symbols. Color code as in Fig. 2 D, with a column for OS current in Na^+^ and K^+^ added (dark yellow). Note the large ouabain-inhibited current in Na^+^ (yellow) and an even larger in Na^+^ with K^+^ (dark yellow) due to K^+^ stimulation of ouabain binding in this mutant (see text). K^+^ binding in ouabain is less evident at −20 mV.

Because these two mutations are located in M3, close to the ouabain-binding cavity (Fig. 1), we tested whether the ouabain insensitivity of the inward currents could reflect further reduction of ouabain-binding affinity. We inserted the L302R and G303R mutations in the OS α1 template and repeated similar experiments in oocytes injected with these constructs (Fig. 6). Ouabain inhibits a similar fraction of the Na^+^-dependent current through OS-L302R as 10 mM did on the OR clone (Fig. 6, A and B). In contrast, ouabain inhibited ∼60% of the Na^+^-induced current through the OS-G303R (Fig. 6, C and D). Fig. 6 D also illustrates that K^+^ application in the presence of ouabain completely eliminates the inward current. It is important to note that this K^+^ effect is absent at −20 mV (Fig. 6 C), where the leak has a much smaller amplitude than at −60 mV. This observation confirms that K^+^ actually reduces the inward leak in the presence of ouabain, instead of increasing the outward NKA-cycling current. Thus, in this variant, K^+^ favors ouabain binding instead of the well-known K^+^-ouabain antagonism of WT NKA (Cornelius et al., 2013).

**Figure 7.**
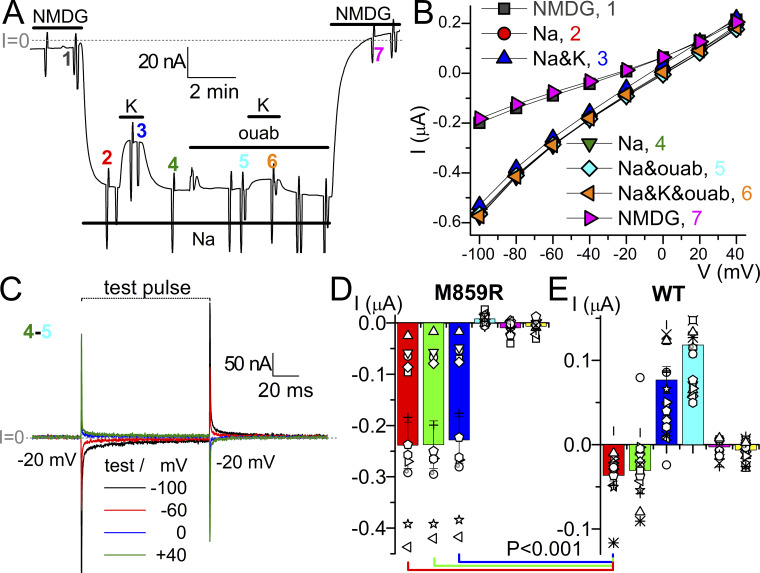
**Steady-state current for OR-M859R-α1β1. (A)** An oocyte injected with OR-M859Rα1β1 held at −20 mV displayed inward current when switching to Na^+^ solution. Application of 4.5 mM K^+^-activated outward current. The holding current was not altered by application of 10 mM ouabain but the inhibitor prevented activation of current by K^+^ application (the remnant ∼5 nA of current induced by K^+^ at this voltage probably reflect current through K^+^ channels due to voltage-dependent release of Ba^2+^ block; Armstrong et al., 1982). Return to NMDG demonstrates stability of the oocyte throughout the experiment. **(B)** I–V curves in different external solutions at the times indicated in A. **(C)** Ouabain-inhibited current elicited by pulses at the indicated voltages. **(D)** Mean difference-currents at −60 mV overlapped with all individual experiments as open symbols. **(E)** Bar graph with the equivalent subtractions in control WT-injected oocytes measured on the same days as those in D. Horizontal lines indicate the mean difference-currents with significantly larger inward current than the Na^+^ minus NMDG subtraction, the largest inward current from WT oocytes (*t* test). Bar graph color code as in Fig. 3 D.

The characteristics of the OR-M859R-α1β1 are shown in Fig. 7. A recording at −20 mV of an OR-M859R oocyte, 7 days after injection (same day as the WT oocyte shown in Fig. 3 A), illustrates the much lower expression of this disease variant (Fig. 7 A). After replacing NMDG with Na^+^ caused a large inward current deflection, K^+^ reversibly activated 31 nA of outward current. Ouabain application had no effect on the holding current, but its presence prevented NKA current activation by K^+^ (demonstrating NKA cycling function of this poorly expressing variant). The steady-state I–V curves illustrate the current in each condition at different voltages (Fig. 7 B). The OS transient currents (Fig. 7 C) are much smaller than WT oocytes (Fig. 3 C) and also lack steady-state currents at negative voltages. The bar graphs in Fig. 7 D illustrate that oocytes with more than 10 nA of K^+^-induced current constitute a small fraction of the total oocytes (cyan bar), but that the inward currents in all Na^+^ containing solutions (Na^+^-NMDG, red, Na with ouabain–NMDG, green, and Na^+^ and K–NMDG, blue bars) are larger than the Na^+^-induced current in WT oocytes on the same date (Fig. 7 E, red bar).

**Figure 8.**
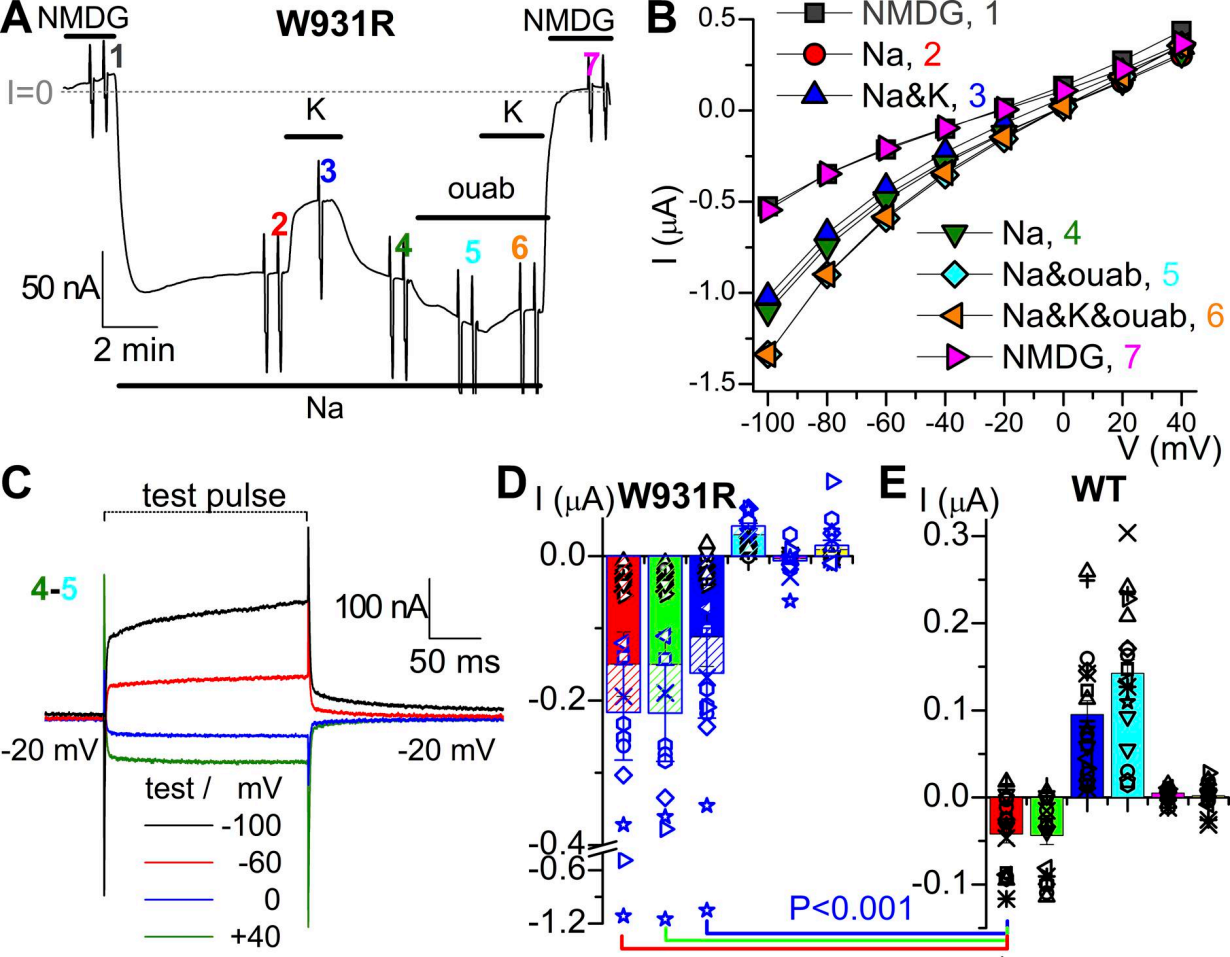
**Electrophysiological characteristics of OR-W931R-α1β1. (A)** Recording from an oocyte at −20 mV. Switching from NMDG to Na^+^-induced inward current. Application of 4.5 mM K^+^ in Na^+^ solution activated NKA current. Ouabain caused an inward shift on the holding current and inhibited subsequent activation of outward current by K^+^. Return to NMDG shows stability of the oocyte. **(B)** I–V curves obtained in different external solutions at the times indicated in A. **(C)** OS current elicited by 200-ms pulses to the indicated voltages showing the increase in current induced by the NKA inhibitor. Note that negative voltages cause ouabain-dependent slow activation of inward current that did not reach steady state after 200 ms at −100 mV (black line). **(D)** Mean difference-currents at −60 mV overlapped with symbols for individual experiments. Blue symbols indicate oocytes in which ≥25 nA K^+^-induced current was observed. These highly expressing oocytes were averaged in the open patterned bar graphs. **(E)** Bar graph with the equivalent subtractions in control WT-injected oocytes measured on the same dates as those in D. Horizontal lines indicate the mean difference-currents with significantly larger inward current than the Na^+^ minus NMDG subtraction, the largest inward current from WT oocytes (*t* test). Bar graph color code as in Fig. 3 D.

Currents in oocytes injected with the OR W931R-α1β1 are shown in Fig. 8. The representative experiment (Fig. 8 A) illustrates that switching to Na^+^ caused a large inward current deflection at all voltages and that K^+^ application activated 50 nA of outward current. This K^+^-activated current was observed at all voltages (Fig. 8 B). Ouabain application in Na^+^ solution increased inward current (Fig. 8, A−C) and prevented activation of NKA current by K^+^ (Fig. 8, A and B). The subtracted current (Na^+^–Na^+^ with ouabain) in response to 200-ms-long pulses illustrates that ouabain increases the current (Fig. 8 C, seen as outward at negative voltages and inward at positive ones). This effect is enhanced at negative voltages and not fully completed after 200 ms at −100 mV. The solid-colored bars in Fig. 8 D are the average of all the experiments, while the patterned bars show the mean of experiments with more than 25 nA of K^+^-induced current, which are identified as blue symbols. The larger the K^+^-induced current, the larger inward current in Na^+^ solutions. Furthermore, the small yellow bar on the right illustrates that ouabain activates, instead of inhibiting, the leak through this mutant, as will become more evident in the next section.

### Charge carriers of the passive current

**Figure 9.**
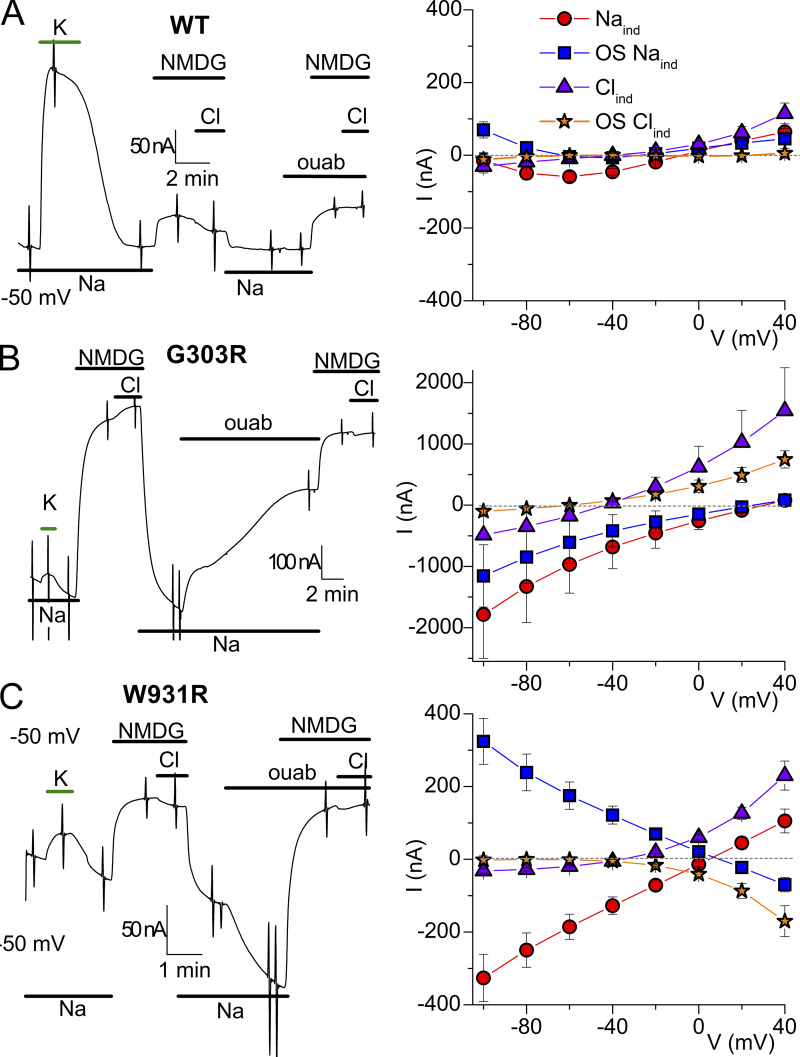
**Ion selectivity of leak currents. (A)** Left, an OS-WT-α1β1 oocyte held at −50 mV in 125 mM Na^+^. Application of 4.5 mM K^+^ reversible activates outward current. Substituting Na^+^ with NMDG-MS and subsequently with NMDG-Cl caused minor changes. After Na^+^ reapplication, 1 mM ouabain was added, then NMDG-MS, and finally NMDG-Cl with ouabain. Right, mean I–V curves (*n* = 9) showing the Na^+^-induced current (Na^+^ minus NMDG-MS, Na_ind_, red circles), the OS Na-induced ([Na^+^ minus NMDG-MS in ouabain], OS Na_ind_, blue squares), the Cl^−^-induced (NMGD-Cl minus NMDG-MS, Cl_ind_, violet triangles), and the OS, Cl^−^-induced currents ([NMDG-Cl minus NMDG-MS in ouabain], OS Cl_ind_, orange stars). **(B)** Left, an OS-G303R oocyte held at −50 mV application of 4.5 mM K^+^ in 125 mM Na^+^ activated only endogenous NKA. Switching to NMDG-MS caused a large outward current shift. Replacing NMDG-MS with NMDG-Cl caused further outward current. Upon returning to Na^+^, ouabain drastically reduced inward current. NMDG-MS and NMDG-Cl were then applied in the presence of ouabain. No outward current was activated by Cl^−^ in the presence of ouabain. Right, mean difference I–V curves as in A (*n* = 5). **(C)** Left, an OS-W931R oocyte displays an inward current drift in 125 mM Na^+^. K^+^ application activated a mix of endogenous and exogenous NKA. A switch to NMDG-MS caused outward current deflection. NMDG-Cl triggered small changes in holding current. Reapplication of Na^+^ resumed the inward current drift. The drift rate was accelerated by 1 mM ouabain. Switching to NMDG-MS in the presence of ouabain brought the current back to the level of the first NMDG-MS application, demonstrating oocyte stability. Substituting NMDG-MS with NMDG-Cl in the presence of ouabain caused a smaller inward current deflection than before ouabain application. Right, mean difference I–V curves (*n* = 4).

Figs. 4, 5, 6, 7, and 8 demonstrate that all four variants carry significantly more inward current than WT oocytes at negative voltages. We reasoned that the positively charged arginine of the variants may open an alternative ion pathway and perhaps also facilitate permeation of small anions. We designed experiments to test whether Cl^−^ permeates through the OS versions of G303R and W931R, the only two variants with leak currents altered by ouabain (Fig. 9). Experiments are shown with a representative trace at −50 mV on the left and the indicated subtracted I–V curves on the right. Fig. 9 A shows the OS-WT-α1β1 control and the maneuver used with all constructs. Following activation of NKA by application of K^+^, the 125 mM Na^+^ solution was substituted by NMDG. Subsequently, MS (the main anion) was substituted by Cl^−^ (NMDG-Cl). Then the solution was returned to Na^+^, where ouabain was applied. Finally, the standard NMDG-MS and then NMDG-Cl were applied in the presence of ouabain.

The difference I–V plots shown are Na^+^-induced (Na^+^ minus NMDG-MS, red circles), the Na^+^-induced current sensitive to ouabain (Na^+^-induced current before ouabain minus Na^+^-induced current after ouabain, blue squares), the Cl^−^-induced current (NMDG-Cl minus NMDG-MS, violet triangles), and the OS Cl^−^-induced current (Cl^−^-induced current before ouabain minus Cl^−^-induced current after ouabain, orange stars). There is more inward current in NMDG-MS than in Na^+^ at potentials less than or equal to −80 mV) due to H^+^ permeation through WT pumps in the absence of Na^+^ and K^+^ (Mitchell et al., 2014; Vasilyev et al., 2004), but there is no OS Cl^−^-dependent outward current at positive voltages.

Fig. 9 B shows the results with OS-G303R-α1β1 oocytes. Substitution of Na^+^ with NMDG caused a large outward current deflection. Replacement of MS with Cl^−^ causes a small outward shift in the current at −50 mV (a potential that will inhibit Cl^−^ inflow). Upon returning to Na^+^, ouabain reduced the inward current. Ouabain’s presence also reduced the small outward current observed when switching from NMDG-MS to NMDG-Cl. In the right panels, the Na^+^-induced current (red circles) was partially inhibited by ouabain application (ouabain-sensitive current, blue squares), as in Fig. 6. The Cl^−^-induced current (violet triangles) is significantly larger than in control oocytes expressing OS WT α1. The OS Cl^−^-induced current (orange stars) is outward at voltages above −60 mV, demonstrating Cl^−^ permeability (as inward flow of Cl^−^ causes an outward current). Note that the OS currents induced by Na^+^ at −40 mV and by Cl^−^ at +40 mV have nearly identical amplitudes (in opposite direction), indicating similar permeability rates.

Fig. 9 C shows the same type of experiment with the OS-W931R-α1β1. The continuous trace illustrates that the current drifts in Na^+^ solution in a way reminiscent of deteriorating oocytes. However, the switch to NMDG arrested the drift, causing an outward deflection toward a stable current. The current drift resumed when Na^+^ was reapplied and was accelerated by ouabain, stabilizing at a new level. This observation suggests that the most permeant state is an E2P state (Fig. S1), with the drift probably reflecting the increase in Na^+^ concentration driving phosphorylation of the variant (which has reduced Na^+^ affinity, as shown in the next section). The OS I–V curves (blue squares for Na^+^, orange stars for Cl^−^) run in opposite direction to the ion-induced ones (red circles for Na^+^, violet triangles for Cl^−^) because ouabain nearly doubled the currents, rather than reducing them. Again, note the nearly identical absolute value of the Na^+^- and Cl^−^-induced OS currents at −40 mV and +40 mV, respectively. It should be noted that due to the slow drift observed that probably reflects the slow buildup of Na^+^ driving phosphorylation of the pumps, the effect of ouabain might disappear if the oocyte were allowed to reach steady state in Na^+^ (see the smaller average effect of ouabain on Na^+^ solutions in the experiments with the OR clone in Fig. 8). If this maximum would have been achieved before addition of ouabain, the OS Cl^−^ permeability would not have been detected because all the NKAs had been in the permeating states in the first Cl^−^ application.

### Enzymatic characteristics of W931R

The enzymatic properties of L302R, G303R, and M859R were previously reported (Schlingmann et al., 2018). Due to the very low activity of the mutants, only Na^+^-dependent phosphorylation and its sensitivity to the presence of K^+^ were measured. The apparent affinity for both Na^+^- and K^+^-driven reactions were reduced in L302P, while the kinetic effect of the G303R and M859R appears to reflect a reduced stoichiometry for the interaction with either ion (Schlingmann et al., 2018). To address the affinity for Na^+^ of W931R, we compared OR-α1 WT and W931R function following transfection of COS-1 cells (see Materials and methods). First, cell growth in the presence of micromolar ouabain (to inhibit the OS endogenous COS-1 cell NKA) requires expression of an active exogenous ouabain-resistant NKA in the PM (Jewell-Motz and Lingrel, 1993; Vilsen, 1992), whose activity must be at least 5% of that of a transfected WT (Holm et al., 2016). Contrasting with the findings by Ygberg et al. (2021), and in keeping with our survival data in Fig. 2, the W931R variant has enough Na^+^ and K^+^ transport activity to allow us growing stable cell lines with the expression level of the active exogenous enzyme being upregulated during growth in the presence of ouabain.

**Figure 10.**
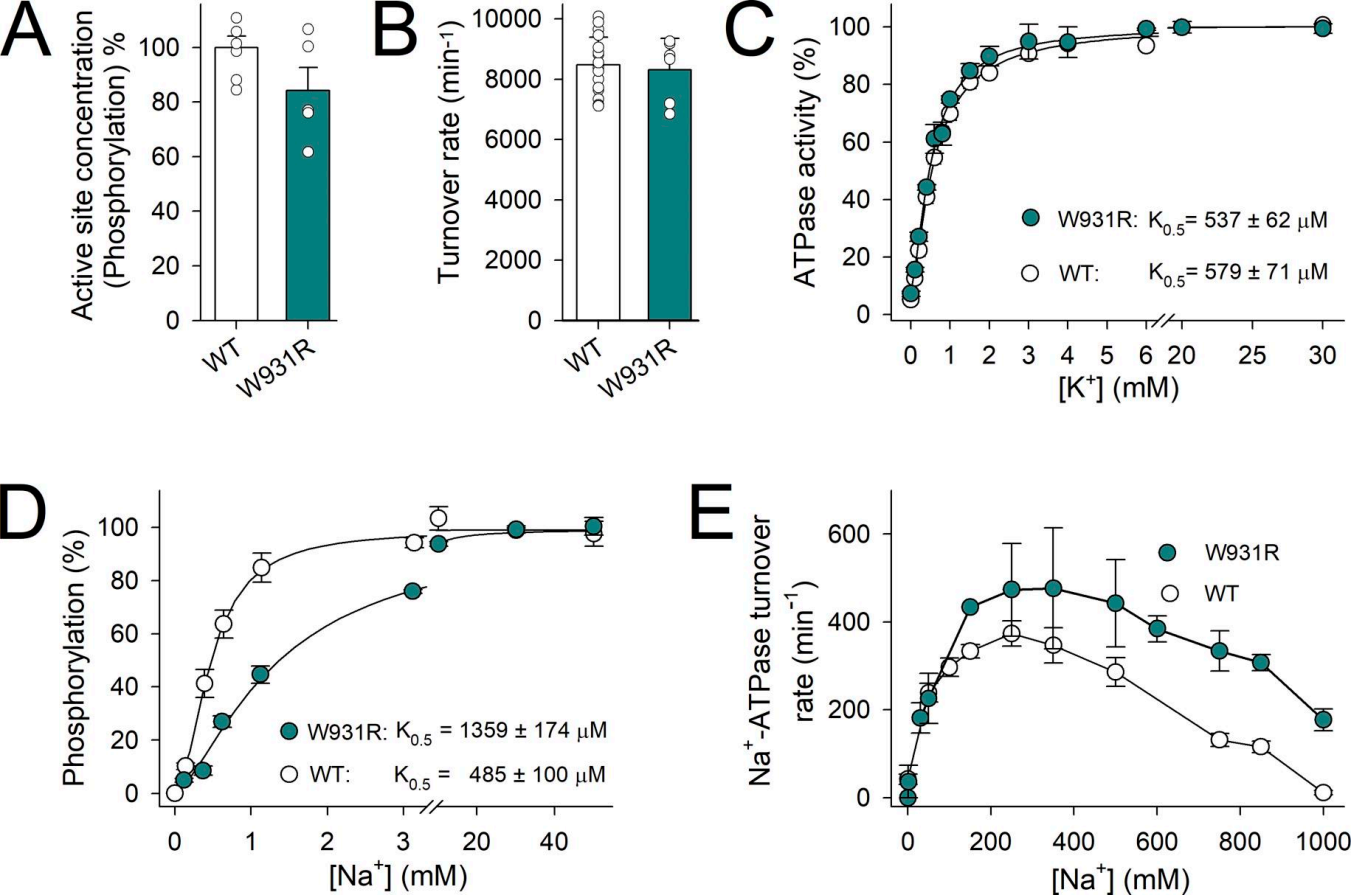
**Enzymatic activity of W931R. (A)** Maximum capacity for phosphorylation from [γ-^32^P]ATP per mg total membrane protein; the active site concentration is presented as % of the WT (36.85 ± 1.6 pmol/mg, SEM, *n* = 6). **(B)** Maximum turnover rate (maximum Na^+^,K^+^-ATPase activity per active site). In panels A and B, the data points from individual determinations are shown superimposed on bar graphs showing mean values with error bars indicating SD. **(C)** K^+^ dependence of Na^+^,K^+^-ATPase activity determined at 40 mM Na^+^. **(D)** Na^+^ concentration dependence of the phosphorylation from [γ-^32^P]ATP. **(E)** Na^+^ dependence of ATPase activity in the absence of K^+^ (“Na^+^-ATPase activity”) shown as turnover rate (Na^+^-ATPase activity per active site). In panels C−E, the data points with error bars represent mean values with SEM (*n* = 3−6). In panels C and D, line plots represent the best fit of a Hill function to the data. The extracted K_0.5_ values are indicated with SD.

Fig. 10 compares the enzymatic properties of OR-W931R with those of OR-WT on an isolated PM fraction. The maximum capacity for phosphorylation from [γ-^32^P]ATP per mg total membrane protein (active site concentration, reflecting the expression level in the PM) was reduced by ∼20% relative to WT (Fig. 10 A), while the maximum turnover rate (maximum NKA activity per active site [Fig. 10 B]) and the apparent K^+^-affinity for NKA activation (Fig. 10 C) were WT-like. The apparent affinity for Na^+^ at the cytoplasmic-facing sites was evaluated by determining the Na^+^ concentration dependence of phosphorylation from [γ-^32^P]ATP in the absence of K^+^ (Fig. 10 D). There is a moderate ∼3-fold reduction of the apparent affinity for Na^+^ caused by the W931R mutation. The reduction in apparent affinity would be larger in the more physiological condition in the cell, where competition between Na^+^ and K^+^ occurs under cycling conditions (because K^+^ binding is not affected by the substitution). There are three ion transport sites in the NKA. Site I and site II interact with either Na^+^ or K^+^, in a conformation dependent manner, while site III only interacts with Na^+^ (Kanai et al., 2013; Kanai et al., 2023). To address the function of the Na^+^ sites in their extracellular-facing configuration, we measured the “Na^+^-ATPase” activity in the presence of varying concentration of Na^+^ without K^+^ (Fig. 10 E). Under these conditions, Na^+^ binds to intracellular-facing sites promoting phosphorylation but also substitutes for K^+^ at the extracellular-facing sites I and II to promote dephosphorylation at a lower rate and lower affinity compared with K^+^. In the absence of K^+^, these effects can be seen as Na^+^ stimulation of the ATPase activity over a range of Na^+^ concentrations. However, extracellular Na^+^ binding to site III also shifts the E_1_P ↔ E_2_P transition toward E_1_P causing inhibition of Na^+^-ATPase activity (backward reaction, rebinding external Na^+^ in Fig. S1), evident at high Na^+^ concentrations for the WT. This inhibition occurred with a reduced apparent affinity in W931R, revealing a minor defect of site III in its extracellular-facing configuration. Thus, the combined results of Fig. 10, D and E, suggest that the Na^+^ affinity of site III is reduced, irrespective of whether Na^+^ binds to this site from the cytoplasmic side or the extracellular side of the membrane.

### Cryo-EM structure of W931R

**Figure 11.**
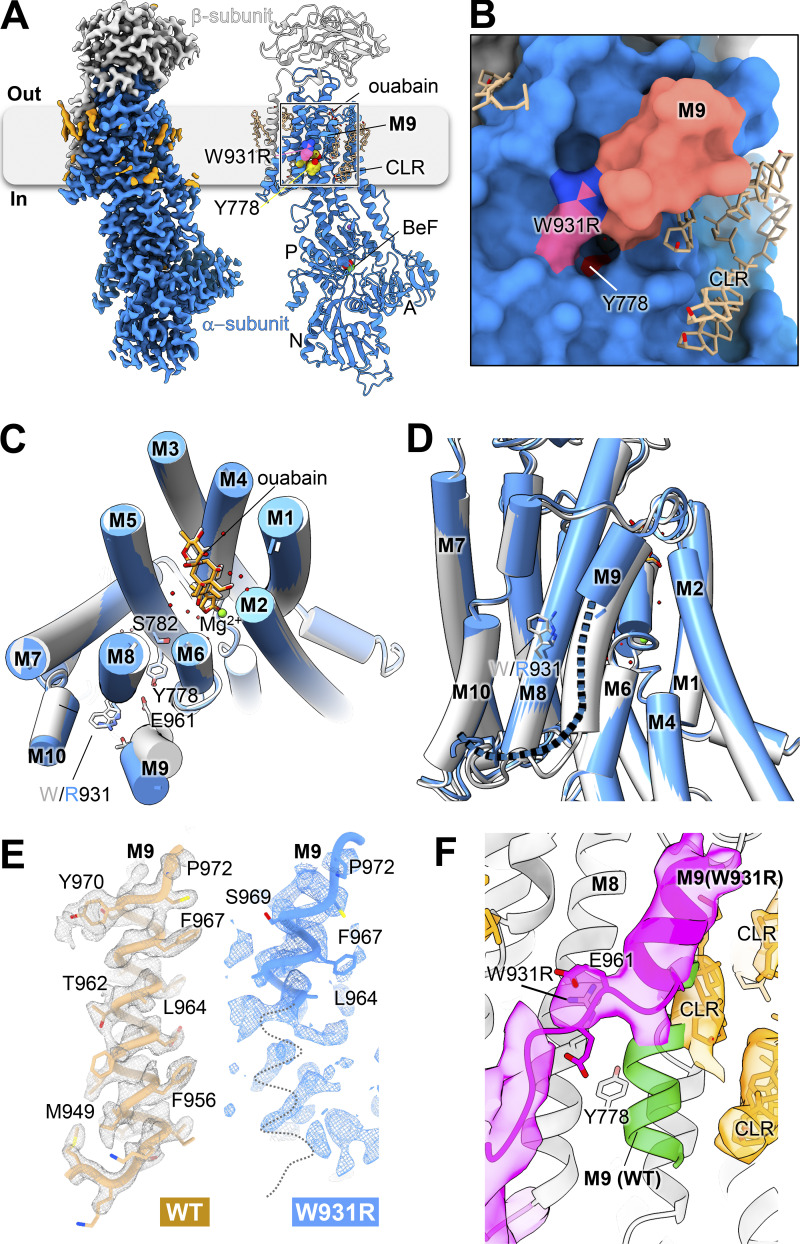
**W931R-α1β1 cryo-EM structure in the ouabain-E2P state. (A)** Left, side view of the EM density map. Orange densities probably represent bound cholesterol (CLR). Right, cartoon model with W931R (magenta) and Tyr778 (yellow). **(B)** Zoomed-in surface representation around W931R. Only the extracellular part of M9 (salmon) could be modeled. The oxygen atom of Tyr778 illustrates exposure of site III due to distorted M9. **(C and D)** Overlap of the transmembrane regions of WT-α1β1 (gray) and W931R-α1β1 structures in the ouabain-bound E2P conformation, viewed from the extracellular side (C) and parallel to the membrane (D). The M9 disordered region of the W931R illustrated by a dotted line; the β subunits were removed for clarity. **(E)** Sharpened EM density maps of M9 superimposed with the cartoon models for WT (*left,* wheat) and W931R (*right*, blue). The dotted line represents a chain trace of the WT M9 when superimposed. **(F)** Details around M9. The magenta surface represents the unsharpened EM density map 4 Å from M9, and the orange one shows cholesterol (CLR). The green cartoon shows the superimposed M9 helix from WT.

We solved the cryo-EM structure of the OS human W931R-α1β1 with beryllium fluoride (E2P state) and bound ouabain (Fig. 11). Fig. 11 A shows the EM density map (*left*) and cartoon model *(right*), highlighting the mutated Arg931 in magenta, as well as three residues essential to form site III, in yellow: Tyr778 (Spontarelli et al., 2022), Ser782 (Nielsen et al., 2024), and Asp933 (Einholm et al., 2010; Moreno et al., 2022). The zoomed-in surface representation around W931R (Fig. 11 B) illustrates the exposure of the side-chain oxygen of Tyr778 (red) to the protein surface. As a control, we also solved the WT structure without FXYD in the same ouabain-bound E2P conformation. The transmembrane domains of WT (light gray) and W931R (blue) are superimposed in Fig. 11, C and D. Ouabain binding and Mg^2+^ coordination within the cation-binding sites are practically identical in both structures (Fig. S2, RMSD = 0.857 Å), but the intracellular half of M9 is disordered in W931R (Fig. 11, D and E). The unsharpened EM density map allowed chain tracing of W931R’s M9, revealing that its course deviates significantly from the single helical structure observed in WT (Fig. 11 F), kinking right at Glu961 a crucial residue for site III’s structural integrity in the WT (Young et al., 2022). This explains the reduction in Na^+^ affinity of W931R (Fig. 10). The weakened EM density observed for M9 of W931R variant is attributable to its disorder, and SDS-PAGE analysis of purified sample confirmed that it is not caused by proteolytic cleavage of the protein. This indicates that the occupancy of M9 in its native helical position—where it normally occludes site III—is reduced and that the polypeptide in this region adopts alternative positions.

In the ouabain-bound W931R structure, disordering of M9 exposes the side chain of Tyr778. As a consequence, the structure appears to form an opening from site III toward the cytoplasmic side (Fig. S2 B, arrowhead). Concomitantly, the extracellular pathway is blocked by ouabain. Even in the absence of inhibitor, the pathway would be constrained near site I (Fig. S2). We therefore focused on the region surrounding W931R to investigate the structural cause of the leak current.

**Figure 12.**
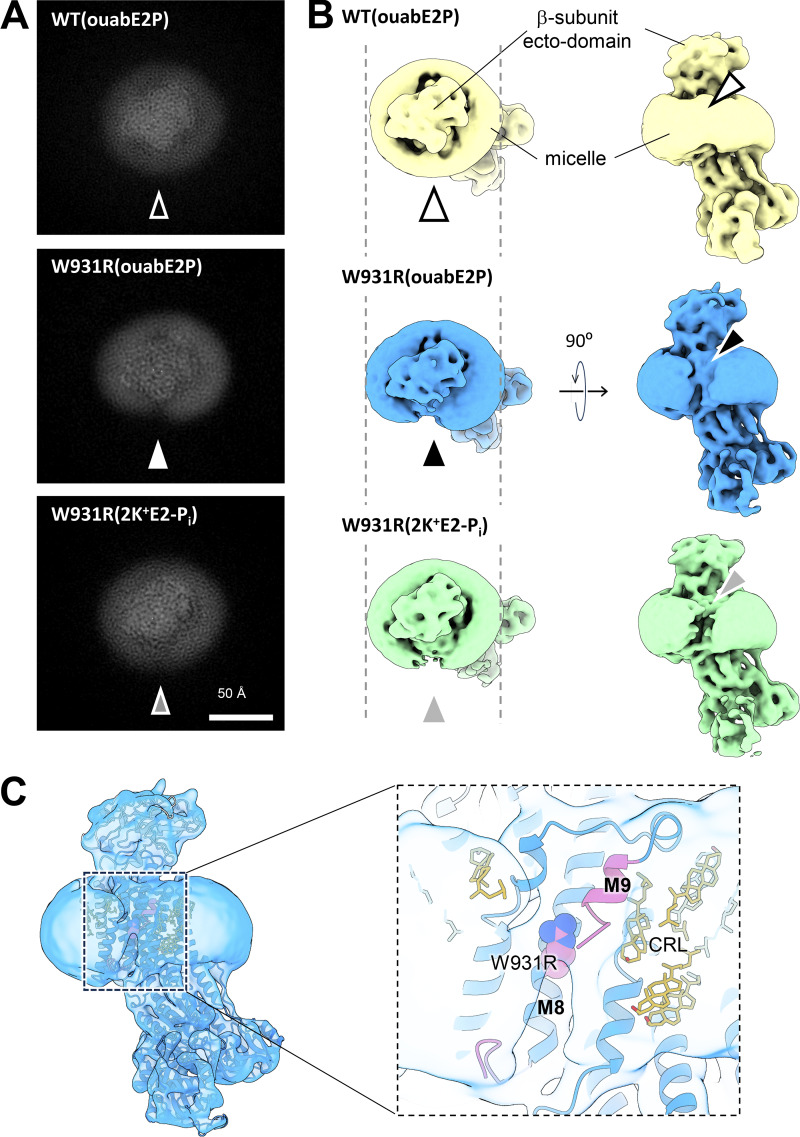
**Altered micelle structure in W931R-α1β1. (A)** Grayscale volume representation of the final reconstruction of WT ouabain-bound E2P state (top), W931R mutant in ouabain-bound E2P state (middle), and K^+^-occluded E2-P_i_ state (bottom), from the viewpoint of the extracellular side showing uneven micelle structure on M9 side (arrowhead), clearly visible in the ouabain-bound structure. Scale bar, 50 Å. **(B)** The Gaussian filtered maps viewed from extracellular side (left) and parallel to the membrane plane (right) evidence the distorted micelle in the E2P-ouabain (middle) and the (2K^+^)E2P_i_ (bottom) structures of W931R. **(C)** Close-up view of the micelle defect at the position of W931R (indicated) in the ouabain-bound E2P state. Gaussian-filtered map (transparent blue surface) is superimposed to its cartoon model (left) and its close-up of indicated position (right). W931R side chain and M9 helix are indicated as pink spheres and pink cartoon, respectively.

Fig. 12 illustrates that the most striking structural feature was found in the surrounding micelle, which appears as an oval shape surrounding the transmembrane region of the WT in the grayscale projection image (Fig. 12 A, *top*), as typically observed for other P-type ATPases and many other membrane proteins. In contrast, the W931R variant displays a region of markedly reduced micelle density (Fig. 12 A, *middle*). As the micelle region of the cryo-EM sharpened map appears particularly noisy, we applied Gaussian filtering (smoothing) to highlight its overall shape (Fig. 12 B). At contour levels adjusted to equalize micelle diameter, a prominent loss of density is evident specifically in the W931R structure. This missing region corresponds to the location where the W931R side chain is exposed and where M9 disordered (Fig. 11 C). Although a reduction in density does not necessarily indicate absence of the detergent, it does suggest that the distribution of the lipid-mimetic, detergent molecules is reduced relative to what is observed in WT at the same position.

We also analyzed the K^+^-occluded E2-P_i_ state of W931R (Fig. 12 A, *bottom,*Fig. S3). An ion pathway within the protein structure was also absent for this K^+^-occluded form (Fig. S3 B). Although the micelle defect is less clear in the grayscale projection image of the K^+^-occluded structure (Fig. 12 A, *bottom*), the Gaussian-filtered map again reveals the micelle distortion at the location of W931R. Notably, in the K^+^-occluded structure M9 is as well resolved as in the WT structure (Fig. S3 C). This indicates that the micelle defect may arise from the arginine side chain exposed to the hydrophobic core of the micelle instead of the disordered M9. Of note, the disordered micelle is observed in both structures, while intraprotein ion pathways that could explain the leak currents are absent from both W931R structures (Fig. S2 and Fig. S3).

### Cellular localization of ATP1A1 variants

**Figure 13.**
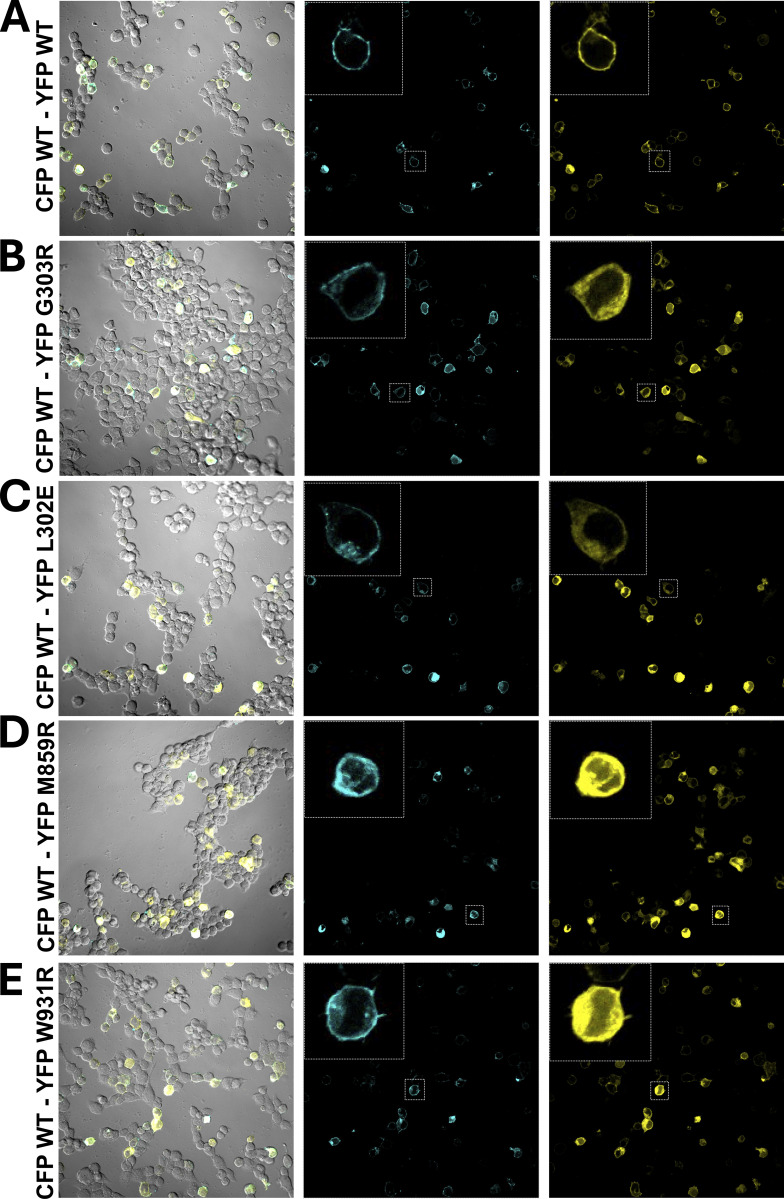
**Localization of α1-hypomagnesemia variants and WT in the same cell.** Images of HEK293 cells co-transfected with CFP-tagged and YFP-tagged α1 (Materials and methods) showing the overlay with transmission detector (left), CFP signal (center), and YFP signal (right). **(A−E)** Co-transfections were CFP-WT-α1 and YFP-WT-α1 (A), CFP-WT-α1 and YFP-L302R-α1 (B), CFP-WT-α1 and YFP-G303R-α1 (C), CFP-WT-α1 and YFP-M859R-α1 (D), and CFP-WT-α1 and YFP-W931R-α1 (E). The top left corners of both color channels signals show a zoomed-in cell.

In oocytes all disease variants showed smaller NKA current than WT (Figs. 3 and 5). In the patients, half of the α1 will be produced by the WT allele and the other half from the variant allele. To evaluate how the disease variants and WT protein localize within the same mammalian cell, we utilized fluorescence microscopy of HEK293 cells (Fig. 13). The cells were co-transfected with a CFP-tagged WT α1 and a YFP-tagged variant α1 accompanied with the β1 subunit (Materials and methods). The idea of this experiment that we have used recently with a CMT variant (Spontarelli Fruit et al., 2025) is that we can both observe the mistargeting of the variant and, concomitantly, determine if the presence of the variant alters localization of the WT subunit (in search for a possible dominant negative effect). The control for these experiments is a transfection with two WT α1 subunits, one tagged with CFP and another tagged with YFP (Fig. 13 A), where the prevalent localization of both CFP- and YFP-α1 subunits at the PM is evident. We then transfected WT CFP-α1 with either YFP-L302R-α1 (Fig. 13 B), YFP-G303R-α1 (Fig. 13 C), YFP-M859R-α1 (Fig. 13 D), or YFP-W931R-α1 (Fig. 13 E).

**Figure 14.**
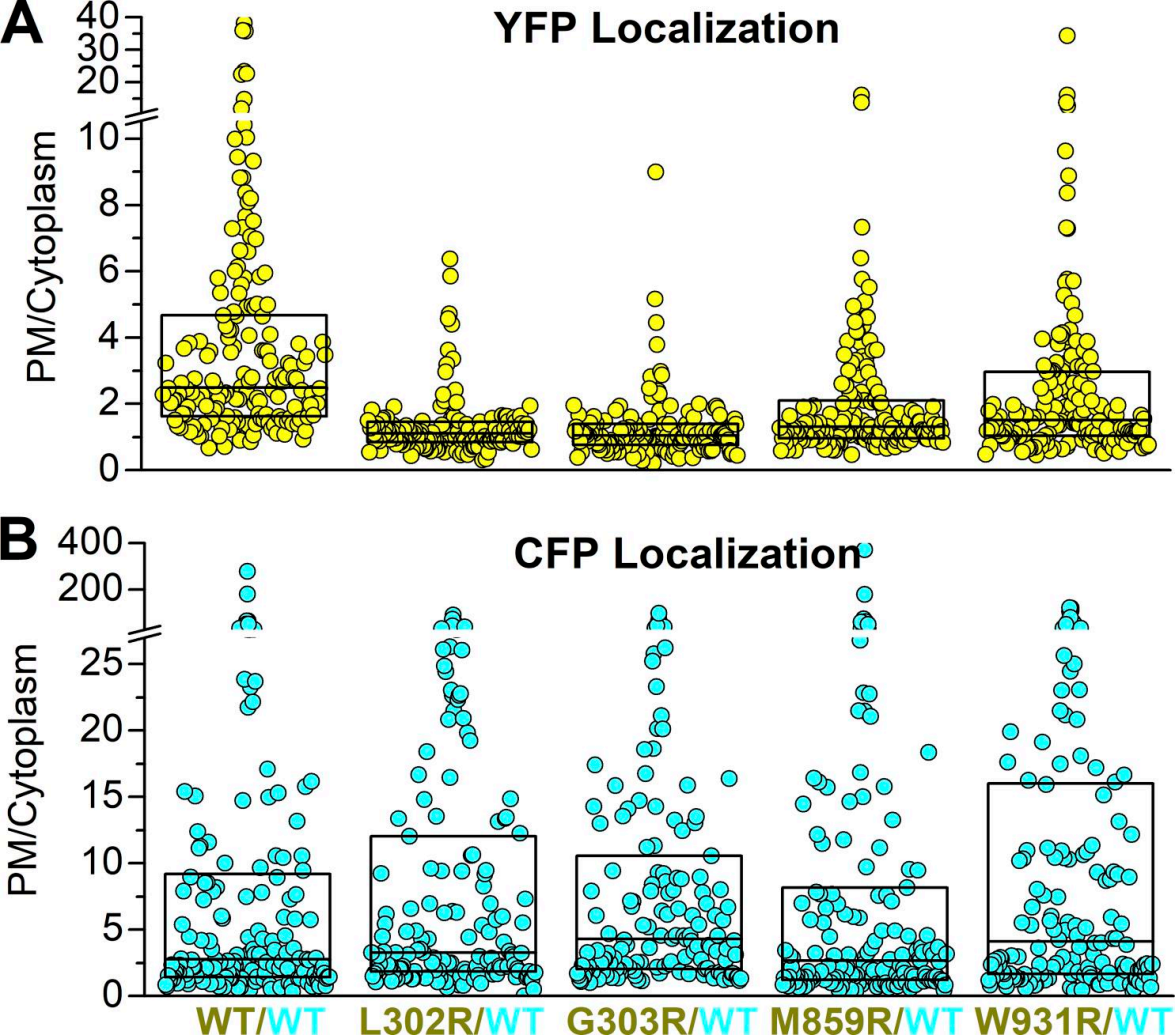
**Analysis of localization of co-transfected variants.** **(A and B) **Fluorescence intensity in the PM/intensity in the cytosol for YFP (A) and CFP (B) for each α1-hypomagnesemia variant and WT co-transfection (Materials and methods). The box goes from the 25th to the 75th percentiles, with the median indicated.

To quantify cellular localization, the fluorescence intensity at the PM was divided by the fluorescence intensity inside the cell (Fig. 14). The whole dataset for both fluorescence proteins illustrates that the YFP-signal for each mutant (Fig. 14 A) is less localized to the PM than the WT YFP. A Kruskal–Wallis statistical comparison confirms that each YFP variant was significantly less localized to the PM than WT. In contrast, the localization ratio for the CFP-WT signal is significantly more scattered for all co-transfections (Fig. 14 B). The Kruskal–Wallis analysis yields a significant difference in the ratio in the CFP signal of M859R with respect to WT and the other three variants (P ≤ 0.05), while the G303R also appears to be higher than WT and M859R (P ≤ 0.05).

## Discussion

Our evaluation of the characteristics of all four α1 variants causing hypomagnesemia with Mg^2+^ supplementation-resistant seizures sheds light onto previously reported observations, providing a plausible common mechanism for development of hypomagnesemia and seizures associated with these variants. The major observations from our study are that all variants (1) have reduced PM expression, which combined with reduced turnover rates due to kinetic effect of the mutation causes a major reduction in NKA activity and (2) carry aberrant passive leak currents. Our meticulous experiments uncovered novel characteristics for each variant and provide the first atomic resolution structure of a leaky disease variant. In what follows, we discuss our observations and conclusions in the context of previous reports.

### Reduced PM insertion of α1 variants

We showed that all four YFP-tagged variants had significantly less PM localization than WT with an apparent retention in intracellular compartments (Figs. 13 and 14). Our results are in keeping with the previously reported reduction of PM expression of W931R in neurons (Ygberg et al., 2021). The L302R and G303R variants showed significantly more intracellular localization than the M859R and W931R. Concomitantly, CFP-tagged WT α1 co-transfected with the YFP variants showed minor differences in localization to those co-transfected with YFP variants. Similar results, albeit with a less drastic reduction in PM localization have been reported for G549R-α1, a variant causing intermediate CMT (Spontarelli Fruit et al., 2025). The large reduction in PM localization suffices to explain the drastic reduction of survival in the ouabain-complementation assay (Fig. 2), even in variants that show some level of electrogenic transport like M859R and W931R. Nevertheless, the extent of expression reduction in native kidney cells and neurons remains unknown, as the overexpression systems miss the native promoters and the cues for intracellular localization of the NKA isoforms in those organs. Being common in both CMT and hypomagnesemia phenotypes, the loss of function of one allele product due to reduced expression at the PM cannot explain the specific phenotype of the four variants evaluated here.

### Functional characteristics of ATP1A1 variants

Compared with WT injected oocytes, oocytes expressing all four disease variants displayed inward currents at physiological voltages, with physiological extracellular concentrations of Na^+^ and K^+^ (Figs. 4, 5, 6, 7, 8, and 9). The presence of large inward currents in L302R- and G303R-injected oocytes bathed by Na^+^ solutions agree with the findings by Schlingmann et al. (2018), who described an external Na^+^-dependent depolarization of mammalian cells expressing it. They also reported a reduction in the IC_50_ for K^+^-dependent inhibition of the Na^+^-dependent phosphorylation. Notably, they did not report Na^+^,K^+^-ATPase activity, which is consistent with the absence of K^+^-induced NKA currents in our experiments. Given that both M3-located variants introduced in the OR template have very large leak currents that cannot be inhibited by 10 mM ouabain and that lack K^+^-induced outward currents (Figs. 4 and 5), the reduced survival rate of cell transfected with them, in the ouabain complementation assay of Fig. 1, is unlikely to represent an increased sensitivity to ouabain of the OR variant. In fact, the passive current through the G303R introduced in the ouabain-sensitive human α1 template was slowly inhibited by ouabain (Figs. 6 and 9), indicating that the disease mutation itself significantly reduced ouabain binding affinity. Ouabain inhibition of leak currents allowed us to demonstrate Cl^−^ permeation through G303R, but not through L302R.

Conclusions regarding M859R were more difficult to corroborate because the variant required longer time after injection to show very small K^+^-activated OS outward currents (Fig. 6), a result in keeping with the reduced Na^+^-dependent phosphoenzyme levels reported by Schlingmann et al. (2018). Importantly, M859R oocytes that displayed clear K^+^-induced, OS current (i.e., expressing oocytes) also presented significantly more inward currents in external physiological solutions that were significantly larger than the Na^+^-induced currents in control oocytes and were not inhibited by ouabain. This result contrasts with the lack of inward currents reported by Schlingman et al. in NCI-H295R cells expressing this variant (Schlingmann et al., 2018), which probably reflects an even lower expression of the mutant in their preparation than in ours. The lack of ouabain effect on the M859R leak indicates that the leak pathway is different from the path for normal active transport, whose external access is inhibited by ouabain, and precluded evaluating Cl^−^ permeability through this poorly expressing variant.

The Na^+^-dependent inward current in oocytes expressing W931R presented a drift, which was evident at −50 mV (the reason for most of our experiments being done holding at −20 mV, to avoid confusion with oocyte deterioration and death). Ygberg et al. observed similar currents in oocytes perfused with external solutions containing small cations (Ygberg et al., 2021). Like our observations at −50 mV (Fig. 9), their reported inward current increased slowly at −70 mV, but appeared unaffected by 10 μM ouabain (cf. Fig. 4, in Ygberg et al., 2021). In our hands, ouabain accelerated stabilization of the leak, indicating that E2P is the configuration showing the most leak current. Therefore, the drift may arise because more negative voltages cause a larger inward current that increases the Na^+^ concentration near the intracellular side of the membrane stimulating phosphorylation of the variant (which has a reduced affinity for intracellular Na^+^), thereby increasing the occupancy of the E2P conformation, carrying a larger leak. In this context, the effect of ouabain would depend on how much intracellular Na^+^ is really present when ouabain is applied. If ouabain were applied after full phosphorylation of the oocytes there would be no increase in leak current. This could explain the lack of ouabain effect on the leak currents in the previous report (Ygberg et al., 2021) and also the difference in the amplitude of the ouabain-induced current between the experiments in Fig. 9 and those averaged in Fig. 8 D. We also found that W931R carries out electrogenic Na^+^/K^+^ transport (Fig. 8) as well as Na^+^- and K^+^-dependent ATPase activity with reduced affinity for intracellular Na^+^ (Fig. 10). While ouabain inhibited the K^+^-induced current, it activated the leak currents. The ouabain effect was seen in both the Na^+^-dependent inward current at negative voltages (Fig. 8 D and Fig. 9) as well as the Cl^−^-dependent outward currents at positive voltages (Fig. 9), a result that suggests that the leak pathway does not strongly select between cations and anions, with Cl^−^ also permeating. By contrast, Ygberg et al. reported that W931R is inactive and unable to bind ouabain (Ygberg et al., 2021).

That ouabain binds to W931R is unequivocally demonstrated by its effect on K^+^-induced currents (Fig. 8) and by the cryo-EM structure (Fig. 11), which shows Mg^2^^+^ and ouabain binding identical to that in WT (Fig. S2). Three lines of evidence demonstrate that W931R has enzymatic activity. First, the survival of HEK293 (Fig. 2) and growth of COS-1 cells transfected with OR W931R in the presence of ouabain; second, K^+^ application activated outward current (Fig. 8), and third, the Na^+^- and K^+^-dependent ATPase activity in membrane preparations with active-site phosphorylation, maximal turnover rate, and K^+^-dependent NKA activity comparable with those in WT (Fig. 10, A−C). The transport capability of W931R is underscored by the cryo-EM structure, showing ability to occlude K^+^ (Fig. S3). A lower transport activity in cells expressing W931R may be caused by a threefold reduced affinity for Na^+^-dependent phosphorylation (Fig. 10 D, reflecting Na^+^ interaction at intracellular sites). This apparently mild reduction would have much more drastic physiological consequences because it would be exacerbated by the presence of K^+^ in the intracellular milieu. Similar reductions in affinity for intracellular Na^+^ have been proposed to underly the pathophysiology of the two non-leaking α1 variants linked to hyperaldosteronism G99R and I327S (Beuschlein et al., 2013; Meyer et al., 2017; Meyer et al., 2019) and has also been found in numerous *ATP1A2* and *ATP1A3* variants causing neurological disorders (Blanco-Arias et al., 2009; Einholm et al., 2010; Friedrich et al., 2016; Holm et al., 2016; Tavraz et al., 2008; Toustrup-Jensen et al., 2014).

### Structure of W931R and the pathway for passive permeation

We present what, to our knowledge, are the first structures of α1β1 NKA without FXYD associated to them. The human WT α1β1 E2P-ouabain structure was very similar to the E2P structures of WT α1β1FXYD2 from pig kidney (PDB 7WYT, Kanai et al., 2022), with the only remarkable difference being the absence of FXYD protein. We also solved the structure of W931R-α1β1 in two conformations: the ouabain-bound, Mg^2+^ occluded E2P (Figs. 11, 12, and S2) and the K^+^ occluded E2(2K^+^)Pi (Fig. 12 and Fig. S3). The structure of the most permeant E2P-ouabain state is drastically altered for transmembrane segment M9, which is kinked right at Glu961. In the E1 conformation with 3 Na^+^ ions occluded of an NKA-mimicking quadruple mutant of the H^+^, K^+^-ATPase, there is a hydrogen bond network between Trp931 in M8, Glu961 in M9, a water molecule, and Tyr778 in M5, stabilizing site III (Young et al., 2022). Thus, W931R must disrupt the same H^+^-bond network, altering the interaction with Na^+^ from both extracellular (reduced inhibition of Na^+^-ATPase activity caused by higher Na^+^ concentrations, Fig. 10 E) and intracellular sides (threefold reduction in affinity for Na^+^-dependent phosphorylation, Fig. 10 D).

The K^+^-occluded structure has a normal M9 (Fig. S3). Common to both structures is a disordered detergent micelle surrounding the protein, causing a concavity where W931R is located (Fig. 14 A). To the best of our knowledge, such disordered micelle has never been described in any previously reported P-type ATPase cryo-EM structure. The micelle disposition is suggestive of a conduit (Fig. 13 D). We speculate that, if present when the membrane protein is in the lipid bilayer, a similar conduit could form the basis for passive ion transport of ions through a pathway involving the protein–membrane interface, instead of through the center of the protein. Whether a similar conduit occurs in a lipid bilayer and with other Arg variants causing OR leak currents will probably require solving the structures of different disease variants in a lipidic membrane using nanodics.

### Passive currents through NKA variants

Passive leak currents through WT, disease variants, and other α1 mutants have been described under different conditions. WT NKAs in the E2P state with ATP bound (Stanley et al., 2016) carry a passive inward proton current at negative voltages when are Na^+^ and K^+^ below saturating concentrations in the external milieu (Rakowski et al., 1991; Vasilyev et al., 2004). Although the current is carried by protons, its analysis is complicated by a biphasic response to application of extracellular Na^+^, where Na^+^ activates the H^+^ leak at low concentrations (up to ∼5 mM Na^+^, depending on the external pH) and inhibits the pathway at higher concentrations (being fully inhibited at ∼50 mM Na^+^) (Mitchell et al., 2014). Thus, a mutation that simultaneously reduces the affinity for activation and inhibition by Na^+^ will increase the H^+^ leak in the presence of external physiological Na^+^ concentrations (Meier et al., 2010; Nielsen et al., 2024; Nielsen et al., 2019; Spontarelli et al., 2022; Yaragatupalli et al., 2009). The leak current is abolished by mutations that drastically impair ion coordination at site III (Poulsen et al., 2010; Vedovato and Gadsby, 2014), indicating that the protons transit a C-terminal pathway from site III to the cytoplasmic side. Some pathogenic variants that reduce affinity for both external Na^+^ and K^+^ appear to increase this proton leak current causing depolarization. This mechanism has been proposed for the S779N-ATP1A2 that causes hypokalemic periodic paralysis by depolarizing skeletal muscle fibers (Sampedro Castaneda et al., 2018) and the D801Y-ATP1A3 that causes rapid onset dystonia parkinsonism and/or alternating hemiplegia of childhood by depolarizing neurons (Isaksen et al., 2017). Many somatic, hyperaldosteronism associated, ATP1A1 variants also carry aberrant Na^+^- and/or H^+^-dependent that depolarize adrenal adenoma cells (Azizan et al., 2013; Meyer et al., 2017; Meyer et al., 2019). All these amino acidic substitutions are located between transmembrane segments, causing leak currents that are cation selective (Na^+^ and in some cases H^+^) and fully inhibited by ouabain (Azizan et al., 2013; Meyer et al., 2017; Meyer et al., 2019).

The leak currents caused by the hypomagnesemia variants are mostly insensitive to ouabain, an inhibitor that binds on the external entrance to the ion-binding sites, blocking extracellular ion access (Fig S2). G303R is partially sensitive to ouabain in Na^+^ only external solution, but it is fully inhibited in the presence of both ouabain and K^+^ (Fig. 6 D). Thus, even though K^+^ does not induce outward current in the absence of ouabain, K^+^ interacts with G303R, because it reduces the leak in the presence of ouabain (Fig. 6 D) and because it promotes dephosphorylation of the enzyme (Schlingman et al., 2018). These results suggest that the ion path of the passive leak differs from the pathway transited by the ions during normal WT active transport. Furthermore, the data obtained with G303R and W931R, the variants in which the leak currents were altered by ouabain, demonstrate that Cl^−^ permeates in these variants (Fig. 9), indicating very different selectivities for passive transport than for active transport (where only Na^+^ and K^+^ interact). Taken together, these results strongly suggest that the hypomagnesemia leak pathways are structurally and mechanistically distinct from other WT and disease leak currents described so far. Moreover, the hypomagnesemia variants are all arginine substitutions around the middle of the hydrophobic core, within the protein–lipid interface, located in M3, M7, or M8 (Fig. 1). Thus, we think that, although the various ion pathways probably occur in the protein–lipid interface surrounding different transmembrane segments, the permeation mechanism may be similar. We propose that the leaking ions negotiate a distorted protein lipid interface similar to the one observed in the protein-micelle structures of W931R. Confirming that the leaks through all four variants are permeant to anions will require electrophysiological studies in other cell types with lower endogenous Cl^−^ permeability, while structures of the disease variants incorporated in nanodiscs membranes will be needed to evaluate if these four mutants alter the bilayer in such manner.

### Nonselective leak current as a phenotype specific characteristic for renal and CNS phenotypes

What makes a ubiquitously expressed variant cause one disease phenotype and not another? First, it is important to note that both heterozygous knockout *Atp1a1*^+/−^ mice (with half the α1 subunits) and a healthy human subject with the early truncation variant Y148* lack hypomagnesemia, seizures, or any other NKA variant–associated disease phenotypes (Spontarelli et al., 2024). This indicates that haploinsufficiency caused by disappearance of the full protein product of one allele cannot explain highly penetrant pathologies and requires a malfunctioning protein product. The most common ATP1A1 disease phenotype is CMT. It is perplexing that most CMT variants exclusively cause peripheral neuropathies without phenotypes affecting other systems, including renal function, despite the essential nature and extremely high expression of the NKA α1 subunit in the kidney. Surprisingly, while being the most functionally studied disease mutations, leak currents or other dominant-negative effect have not been described for any CMT variant, irrespectively of the phenotype severity (Cinarli Yuksel et al., 2023; Lassuthova et al., 2018; Spontarelli Fruit et al., 2025). The aberrant leak currents that we described here provide a disease phenotype-specific dominant-negative mechanism for hypomagnesemia and for magnesium supplementation treatment-resistant seizures.

That *ATP1A1* variant-associated seizures remain upon Mg^2+^ supplementation indicates that these are directly caused by the electrophysiological characteristics of the variant α1 protein expressed in CNS neurons (and not secondary to hypomagnesemia activating AMPA receptors). Even a modest, sustained resting potential depolarization caused by small leak currents through mutant NKAs could lead to repetitive firing and hyperexcitability. This would obviously happen due to the Na^+^-driven inward current that depolarizes the neuron, bringing it closer to its threshold, causing hyperexcitability. However, the Cl^−^ permeability can also be important. The Cl^−^ gradient is an essential determinant of neuronal excitability and mutations in proteins involved in maintenance of such gradient, like the electroneutral KCC2, or its dissipation, like the GABA pentameric ligand-gated Cl^−^ channel, are known to cause many forms of epilepsy (Absalom et al., 2024). Furthermore, gain-of-function mutations that drastically increase affinity of GABAA receptor channels for their agonist have been shown to be more epileptogenic with lower survival, with stronger gain of function causing more seizures and decreased survival (Mohammadi et al., 2024). Thus, the NKA leaking Cl^−^ down its electrochemical gradient could cause similar epileptogenesis in the infants carrying the variant proteins. The nonselective leak current can fully explain the occurrence of Mg^2+^ supplementation treatment-resistant seizures in patients with each of the four variants (Schlingmann et al., 2018; Ygberg et al., 2021).

Requirement of the leak for renal hypomagnesemia is less obvious. Mg^2+^ homeostasis is controlled by a complex mechanism involving absorption in the digestive system followed by excretion and reabsorption by the kidneys. Renal Mg^2+^ reabsorption occurs in the proximal convoluted tubule (10–25%), the thick ascending limb of the loop of Henle (50–70%), and finally in the distal convoluted tubule (DCT, 5–10%). The first two occur through the paracellular pathway and the third through the transcellular route (Brenner, 2000; Vetter and Lohse, 2002; Viering et al., 2017). Thus, both mechanisms of reabsorption require the Na^+^ and K^+^ gradients that are built and maintained by NKA. Despite its importance, most known *ATP1A1* germline pathogenic variants cause neurological disease phenotypes without hypomagnesemia. ATP1A1 variant-linked hypomagnesemia probably arises in the DCT, the nephron segment with highest NKA density and where the final fine tuning of Mg^2+^ reabsorption occurs. Variants of various genes involved in transcellular Mg^2+^ transport in the DCT cause hypomagnesemia as one of the penetrant symptoms, including the apical *TRPM6/7* (entry point of Mg^2+^) and *NCC* (NaCl cotransporter, as well as the basolateral *ATP1A1*, *FXYD2*, and *CNNM2* (a controversial putative Na^+^/Mg exchanger), *ClCNKb* (a Cl^−^ channel), as well as the *KJN10* and *KJN16* (inward rectifier K^+^ channels) (reviewed by de Baaij [2023]).

How the leak currents can lead to hypomagnesemia can be explained by how the KCNJ inward rectifier K^+^ channel controls NCC translocation to the PM via the with-no-lysine (WNK) kinase phosphorylation. WNK is inhibited when the intracellular Cl^−^ concentration is high. Thus, when the plasma K^+^ concentration is reduced, the basolateral membrane is hyperpolarized, causing the intracellular Cl^−^ concentration to fall, increasing NCC insertion. An inward Na^+^ leak through the NKA variants would produce exactly the opposite effect, and this will be exacerbated if the variants permeate Cl^−^. At any rate, Mg^2+^ reabsorption in the DCT depends on a delicate balance between basolateral and luminal membrane potentials (Mayan et al., 2018), which will be largely disturbed by the appearance of a basolateral inward current through the NKA.

It should be noted that all substitutions involve replacing hydrophobic residues with a large positively charged arginine in the transmembrane segments, causing large disruptions to the protein and its surrounding bilayer. Other arginine substitutions in transmembrane domains of NKA subunits are known to cause disease. The somatic adrenal-adenoma α1 variant L104R causes hyperaldosteronisms and ouabain-sensitive leak currents, partially carried by Na^+^ (we are not aware of specific testing for Cl^−^ permeability); the germline α1 variant G864R (in M7, five residues closer to the extracellular side than M859) has been linked to seizures without hypomagnesemia in a 5-mo-old patient (Lin et al., 2021), but its functional characteristics have not been reported. Conversely, substitutions of three of these residues to amino acids other than arginine occur in human population sequencing datasets, with one allele each of M859T, M859I, and G864A in gnomAD v.4.1.0 (Karczewski et al., 2020) and one allele of G303A in the All of Us Public dataset (Denny et al., 2019). Although this must be interpreted with caution due to the lack of phenotype information on the individuals carrying these variants and the known phenotypic variability of sodium-potassium ATPase diseases, it suggests that these substitutions might be less deleterious than arginine, consistent with a recent study evaluating G303A (Seflova et al., 2025).

Similar arginine substitutions in the mostly glial α2 (G301R, equivalent to ATP1A1’s G303R in M3, L809R in M6, and G855R in M7, Prontera et al., 2018) and the neuronal α3 subunits (L292R equivalent to ATP1A1’s Leu302 in M3, and P775R in M5, Vetro et al., 2021) have been reported to cause epileptic seizures, but their electrophysiological characteristics have yet to be evaluated. One of the most common α3 mutations resulting in the neurological disorder alternating hemiplegia of childhood is G947R. This arginine substituent is predicted to point toward Y768 and may destabilize the binding network around Y768, which is equivalent to α1 Y778 (Holm et al., 2016). The α3-G947R variant may therefore result in the same type of disturbance of the protein-lipid interphase as seen here for W931R. Of further note, the G41R variant of the renal γ subunit (Fig. 1) causes familial hypomagnesemia (Meij et al., 2000). Mg^2+^ supplementation alleviates this purely renal phenotype. Mechanistic studies point to misrouting of G41R-γ (Meij et al., 2003; Meij et al., 2000; Sha et al., 2008), but it remains unclear whether leak currents are caused by expression of the variant, as large leak currents were reported with G41R, but also with WT γ subunits (Sha et al., 2008), a result never observed by other laboratories (Béguin et al., 1997; Geering, 2006; Meyer et al., 2020). Further evaluation of the electrophysiological characteristics of these germline NKA subunit variants would shed light on whether Na^+^ and Cl^−^ permeable leak currents are required to cause both seizures and hypomagnesemia phenotypes.

## Supplementary Material

Table S1 shows technical details of the cryo-EM structures for W931R-α1β1 and WT-α1β1 NKAs.

## Data Availability

Data are available in the article itself and its supplementary materials.
