# Supplementary material for: On the mechanism of hypomagnesemia with treatment-resistant seizures caused by variants of the Na+,K+-ATPase α1 subunit (ATP1A1)
Source: J Gen Physiol. 2026 Jun 30;158(5):e202513959. doi: 10.1085/jgp.202513959 (PMC13317510; doi:10.1085/jgp.202513959)
Supplement: Table S1 — shows technical details of the cryo-EM structures for W931R-α1β1 and WT-α1β1 NKAs. [file jgp_202513959_tables1.docx]

**Supplementary Materials**

|  |  | Cryo-EM structure W931R-hα1β1 in the ouabain-bound E2P state | Cryo-EM structure WT-hα1β1 in the ouabain-bound E2P state | Cryo-EM structure W931R-hα1β1 in K^+^-occluded E2-P_i_ state |
| --- | --- | --- | --- | --- |
| PDB ID |  | 9WAK | 9WAJ | 24RC |
| EMDB |  | EMD-65821 | EMD-65820 | EMD-69776 |
|  |  |  |  |  |
| **Data collection** |  |  |  |  |
| Magnification |  | 60,000 | | |
| Voltage (kV) |  | 300 | | |
| Electron exposure (e^-^/Å^2^) |  | 60 | | |
| Defocus range (μm) |  | 0.8-1.8 | | |
| Pixel size (Å/pix) |  | 0.752 | | |
| Symmetry imposed |  | *C1* | | |
| Number of movies |  | 18,025 | 12,950 | 12,850 |
| Number of Initial particles |  | 4,786,625 | 4,554,298 | 5,908,488 |
| Number of Final particles |  | 121,048 | 473,771 | 229,314 |
| Box size (extract/final, pix) |  | 320/450 | 360/450 | 320/450 |
| Map resolution (Å) |  | 2.86 | 2.39 | 2.61 |
| Map sharpening B-factor (Å^2^) |  | -93.5 | -77.0 | -72.5 |
| FSC threshold |  | 0.143 | 0.143 | 0.143 |
|  |  |  |  |  |
| **Refinement** |  |  |  |  |
| Initial model used (PDB) |  | 9WAJ | 7WYT | 9KCI |
| Model resolution (Å) |  | 3.0 | 2.5 | 2.7 |
| FSC threshold |  | 0.5 | 0.5 | 0.5 |
| Model composition |  |  |  |  |
| Non-hydrogen |  | 10,287 | 9,879 | 10218 |
| Protein residues |  | 1,261 | 1252 | 1252 |
| Waters |  | 14 | 25 | 71 |
| Ligands |  | OBN, Na^+^, 2Mg^2+^, 12CLR | OBN, Na^+^, 2Mg^2+^ | ALF, 3K^+^, Mg^2+^, 6PCW, 3CLR |
| B-factor (mean value, Å^2^) |  |  |  |  |
| Protein |  | 85.87 | 28.00 | 56.49 |
| Ligand |  | 104.95 | 20.43 | 70.66 |
| Water |  | 77.79 | 20.54 | 35.04 |
| r.m.s.d |  |  |  |  |
| Bond length (Å) |  | 0.002 | 0.003 | 0.002 |
| Bond angles (°) |  | 0.92 | 0.699 | 0.737 |
| Validation |  |  |  |  |
| MolProbity score |  | 2.03 | 1.84 | 1.37 |
| Clashscore |  | 7.84 | 8.36 | 5.65 |
| Poor rotamers (%) |  | 2.59 | 2.99 | 1.12 |
| Ramachandran plot |  |  |  |  |
| Favored (%) |  | 95.85 | 97.91 | 97.66 |
| Allowed (%) |  | 4.15 | 2.09 | 2.34 |
| Disallowed (%) |  | 0.00 | 0.00 | 0.00 |

**Table S1.** Technical details of the CryoEM structures for W931R-α1β1 and WT-α1β1 NKAs.
